# Mononuclear Copper(I) 3-(2-pyridyl)pyrazole Complexes: The Crucial Role of Phosphine on Photoluminescence

**DOI:** 10.3390/molecules26226869

**Published:** 2021-11-14

**Authors:** Kristina F. Baranova, Aleksei A. Titov, Alexander F. Smol’yakov, Andrey Yu. Chernyadyev, Oleg A. Filippov, Elena S. Shubina

**Affiliations:** 1 A. N. Nesmeyanov Institute of Organoelement Compounds, Russian Academy of Sciences, Vavilov Str., 28, 119991 Moscow, Russia; krisbar99@gmail.com (K.F.B.); rengenhik@gmail.com (A.F.S.); shu@ineos.ac.ru (E.S.S.); 2 Faculty of Chemistry, Lomonosov Moscow State University, 1–3 Leninskie Gory, 119991 Moscow, Russia; 3 Plekhanov Russian University of Economics, Stremyanny per. 36, 117997 Moscow, Russia; 4 A. N. Frumkin Institute of Physical Chemistry and Electrochemistry, Russian Academy of Sciences, Leninsky prosp. 31/4, 199071 Moscow, Russia; chernyadyev@mail.ru

**Keywords:** copper(I), pyrazolate, pyrazole-pyridine, TD-DFT, photoluminescence, volume buried

## Abstract

A series of emissive Cu(I) cationic complexes with 3-(2-pyridyl)-5-phenyl-pyrazole and various phosphines: dppbz (**1**), Xantphos (**2**), DPEPhos (**3**), PPh_3_ (**4**), and BINAP (**5**) were designed and characterized. Complexes obtained exhibit bright yellow-green emission (ca. 520–650 nm) in the solid state with a wide range of QYs (1–78%) and lifetimes (19–119 µs) at 298 K. The photoluminescence efficiency dramatically depends on the phosphine ligand type. The theoretical calculations of buried volumes and excited states explained the emission behavior for **1**–**5** as well as their lifetimes. The bulky and rigid phosphines promote emission efficiency through the stabilization of singlet and triplet excited states.

## 1. Introduction

**Scheme 1:**
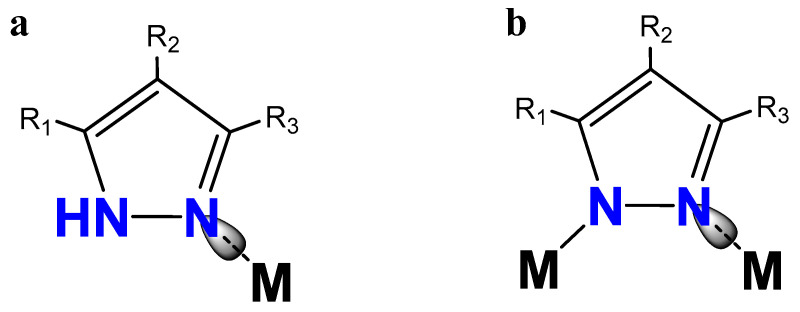
Chemical structures of non-deprotonated pyrazole coordinated to metal via a lone pair of N atom (**a**) and pyrazolate anion in the bridging mode (**b**).

Nitrogen-containing ligands play an important role in the coordination chemistry of group 11 metals. Aromatic amines such as pyrazoles, imidazoles, triazoles, or tetrazoles, which are quite versatile ligands, are popular in this area. Being the Lewis bases, such compounds can act as a monodentate ligand due to the lone electron pair of the nitrogen atom of the imine type. In the deprotonated form, it is a bridging charged ligand, acting as a counterion. Depending on the aim, the reaction conditions are easily variable for creating the target complex. The pyrazoles could be distinguished among this series because of their ease of preparation and rather extensive chemistry [1,2,3]. The presence of two neighboring nitrogen atoms allows obtaining different structures. In the absence of a base, pyrazoles (Pz) coordinate to the metal atoms only via the lone pair of imine-type nitrogen. The non-deprotonated NH group additionally stabilizes the complexes through hydrogen bonds with a counterion or a neighboring molecule (Scheme 1a) [4]. In contrast, the presence of a base or the use of in situ prepared sodium pyrazolate allows obtaining di-, tri-, tetra-nuclear, and polymeric complexes (Scheme 1b) [5,6,7].

Trinuclear coinage metal pyrazolates are most popular due to their planar structures allowing to form stacks via M∙∙∙M or M∙∙∙π intermolecular interactions [8,9]. Compounds of this type possess bright photoluminescence depending on the metal atom, substituents in Pz ligand, and intermolecular interactions [10,11,12,13,14]. From another hand, the presence of the NH group opens up possibilities for ligand modifications. New ligands containing additional coordination centers [15,16], or pyrazole derivatives of borohydride [17,18,19] and methane [20,21,22] could be obtained.

Furthermore, it is possible to distinguish the third possible way for modification of aromatic amines (pyrazole, imidazole, triazole, etc.), which can serve as monodentate ligands in both protonated and deprotonated forms. In this case, the introduction of substituents able to coordinate the metal atom yields the chelating ligands. For this reason, the pyridine-substituted five-membered heterocycles, especially those containing the NH group, gain popularity in various fields of modern chemistry. The photophysical behavior of copper(I) complexes with pyridine-pyrazole/triazole/tetrazole ligands are close to the copper(I) complexes with bipyridine/phenanthroline-type ligands [23,24]. Bipyridine/phenanthroline-type ligands and pyridine-pyrazoles could be considered related but not identical, possessing different chemistry behaviors.

Interaction of deprotonated pyridine-pyrazoles with copper and silver salts leads to the formation of cyclic structures [25]. For the first time, such complexes were identified by Stavropoulos et al. back in 1997 [25]. More recently, Li et al. have shown that similar trinuclear complexes possess efficient, long-lived photoluminescence due to MLCT (metal-to-ligand charge-transfer) transitions of the high-lying ISC (intersystem crossing) pathways [26].

In 2011, a group of Chi, Chou, and Chang described a new type of group 11 metals phosphine complexes with various tailor-made 2-pyridyl pyrrolide chromophores playing a role of counterion [27]. The observed emission is typical for complexes with diimine-type ligands. The effect of a metal atom on the emission was investigated experimentally with the involvement of theoretical studies. Almost simultaneously, several works were published on the study of the photophysical properties of both neutral and cationic complexes with pyridine-amines (pyrazole, triazole, tetrazole, benzimidazole, etc.) [28]. The high efficiency of photoluminescence and the potential for creating solution-processed organic light-emitting diodes have been shown [29,30,31,32,33,34,35,36,37]. On the example of complexes of pyridine-triazole with bis(diphenylphosphino)ferrocene, Chan and Chi et al. have shown the possibility of the formation of both neutral (with ligand in a deprotonated form) and cationic complexes depending on the substituents in a heterocycle [38].

Collins et al. have demonstrated that the cationic copper(I) complex with non-deprotonated pyrazole-pyridine with sulfonamide substituent is a bifunctional catalyst for proton-coupled electron transfer (PCET) processes [39]. Quite recently, Lu et al. reported strongly emissive Cu(I) complexes with pyrazole-methylpyridine and DPEPhos ligand, demonstrating the dependence of the emission properties on the steric and electronic effects of the diimine ligands [40].

**Scheme 2:**
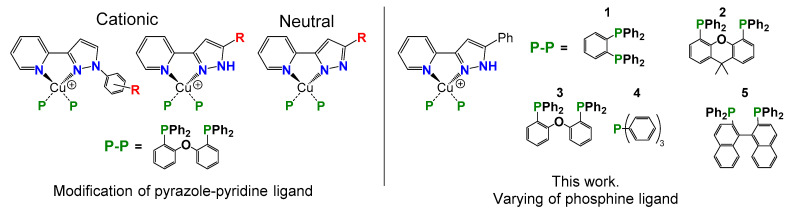
Main paths for structural alteration of Cu(I) heteroleptic pyrazole-pyridine complexes.

All of the above-listed complexes are heteroleptic and contain other ligands coordinated to the metal centers, in particular chelating or bridging bisphosphines. In the case of mononuclear copper complexes, the most often used chelating bisphosphine ligand is DPEPhos (bis(2-(diphenylphosphanyl)phenyl)ether). At that, the influence of phosphorus-containing ligand on the photophysical properties of complexes obtained has not been explored for this class of compounds. Thus far, only the role of the substituent in pyridine or pyrazole fragments has been demonstrated (Scheme 2).

Interestingly, the data on the phosphane tuning are also limited in the case of more popular copper(I) complexes with such diimines as bipyridine and phenanthroline derivatives, [Cu(N^N)(P^P)]^+^. For example, Housecroft et al. have demonstrated the modification of the Xantphos ligand (4,5-bis(diphenylphosphino)-9,9-dimethylxanthene) for application in light-emitting electrochemical cells (LECs) [41]. In 2020 Alkan-Zambada et al. have suggested a theory of the interdependence of buried volume (%V_bur_) and photoluminescence QYs. On the example of 100 heteroleptic copper(I) diimine complexes, they have demonstrated that a high value of V_bur_ is a necessary but insufficient condition for high photoluminescence QY [42]. Quite recently, the effect of the bite angle of the diphosphine ligand on the photoluminescence of the [Cu(N^N)(P^P)]^+^ (N^N = neocuproine) has been studied by Zysman-Colman et al. [43].

The photophysical behavior of copper(I) complexes with pyridine-pyrazole/triazole/tetrazole ligands is close to the copper(I) complexes with bipyridine/phenanthroline-type ligands [23,24]. We should note that bipyridine-type ligands and pyridine-pyrazoles could be considered related but not identical, possessing different chemistry behaviors. The main difference between bipyridine-type ligands and NH-heterocycle-pyridines (pyrazole, imidazole, triazole, etc.) is the ability of the latter to give a deprotonated pyrazolate-anion that not only coordinates to the core metal but also compensates for its positive charge yielding neutral complexes. That becomes the main advantage of using NH-containing heterocycles as ligands, important for the employment of such neutral complexes in fabricating thin films for OLEDs. Surprisingly, this area of research is still not so popular in the case of d^10^ transition metal complexes. For example, the number of heteroleptic Cu(I) pyrazole-pyridine phosphine complexes is quite limited. Only about 30 examples are described so far, including both neutral (deprotonated pyrazole) and cationic (protonated pyrazole) mono- or dinuclear complexes.

Herein we present the synthesis, characterization, and photophysical properties of copper complexes with 2-(5-phenyl-1H-pyrazol-3-yl)pyridine and phosphines: dppbz (1,2-bis(diphenylphosphino)benzene, (**1**), Xantphos (**2**), DPEPhos (**3**), PPh_3_ (**4**), and BINAP (**5**).

## 2. Results and Discussions

### 2.1. Synthesis

Initial 2-(5-phenyl-1H-pyrazol-3-yl)pyridine was synthesized according to the published procedure [44]. The copper complexes (**1**–**5**) were prepared by a two-step reaction. At first, the N^N ligand interacted with an equimolar amount of [Cu(CH_3_CN)_4_]BF_4_ in acetone within one hour. Then to the solution obtained, the appropriate amount of phosphine was added: one equivalent for bisphosphines and two equivalents for PPh_3_. Complexes **1**–**4** were isolated by layering diethyl ether on their dichloromethane solution. In the case of the BINAP ligand, complex **5** precipitated from the reaction mixture and was isolated by filtration. Complexes were characterized by ^1^H and ^31^P NMR spectroscopy, IR, and elemental analyses. The qualitative characteristic of the formation copper complex with a diimine ligand is the presence of ν(NH) stretching vibrations in the IR spectrum. This also demonstrates the absence of ligand deprotonation and the formation of cationic complexes. All complexes obtained are stable under air at ambient conditions.

### 2.2. Crystal Structures

**Figure 1:**
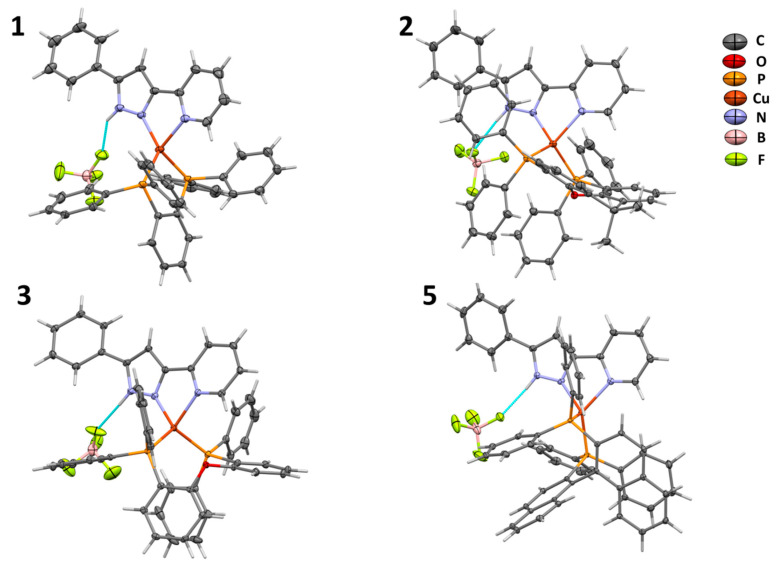
XRD structure of complexes **1**, **2**, **3** and **5** with thermal ellipsoids set at the 30% probability level.

Single crystals of complexes **1**–**3** were grown from CD_2_Cl_2_/hexane solution (*v*/*v* = 1:3), and crystals of complex **5** were obtained by crystallization from acetone/CH_3_CN solution (*v*/*v* = 2:1). Single-crystal XRD analyses reveal that the structures of these complexes (Figure 1) are similar to previously published copper(I) complexes with pyridine-pyrazoles and phosphines. The intramolecular N-H···F(BF_4_) contacts (1.939–2.039 Å) additionally stabilize the complexes’ structure. The coordination of different phosphine ligands to Cu atoms leads to significant differences in the Cu–N bond lengths ranging from 2.001(3) Å in complex **1** to 2.119(3) Å in complex **3**. In contrast, Cu–P bond lengths practically do not depend on the phosphine type (2.232(1)–2.2700(8) Å).

Copper atoms possess tetrahedral geometry with the non-significant variation of N-Cu-N angles in a 79.11(8)°–81.1(1)° range, which is typical for this type of compound. In contrast, the values of P–Cu–P angles significantly depend on the phosphine ligand, being in the range 91.43(4)°–115.08(3)°. Significant differences in P–Cu–P angles lead to the various types of tetrahedral geometry around the copper atom. To compare the geometries of the structures obtained herein and of similar complexes, τ*_δ_* parameters were calculated from the largest (*α*) and second-largest (*β*) L–Cu–L angles as suggested by the Kubiak et al. (Equation (1)) [45]. The τ*_δ_* parameter suggested by Houser et al. [46]. is convenient to use due to the presence of the plier or scissoring type distortions described by Alvarez for bidentate ligands [47].

(1) $$\tau_{\delta }=\frac{\left(360-\left(\alpha +\beta \right)\right)}{141}\times \delta ;\;\delta =\beta /\alpha \;$$

Obtained this way, τ*_δ_* values for complexes **1**, **2**, **3** and **5** are 0.71 0.81, 0.85, and 0.73, respectively. Thus, all complexes obtained are described as distorted tetrahedral complexes (0.63 < τ*_δ_* < 0.9). The changes in τ*_δ_* values can be attributed to different ligand geometries. This also could lead to the differences in the overlapping of copper d orbitals with phosphorus p-orbitals, resulting in a different efficiency of bonding and donation. Moreover, different geometry of copper atoms in complexes **1**–**5** should ultimately affect the stability of the excited states (Supplementary Materials Figure S4). τ*_δ_* values for these complexes and analogous copper(I) pyridine-pyrazoles/triazoles complexes with bisphosphines are presented in Table 1.

### 2.3. Photophysical Properties

**Figure 2:**
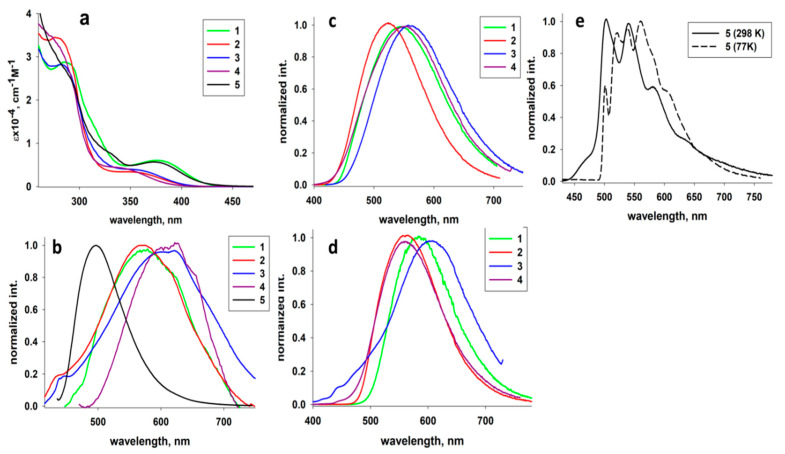
(**a**) UV-vis absorption spectra and (**b**) emission spectra of complexes **1**−**5** in DCE; emission spectra of solid samples **1**−**4** at (**c**) 298 K and (**d**) 77 K; (**e**) emission spectra of solid **5** at 298 and 77 K.

The UV-vis absorption spectra of complexes **1**–**5** were measured in degassed 1,2-dichloroethane (DCE) solution (Figure 2). They exhibit several intense bands in the short wavelength region of the π→π* character, within pyridine-pyrazole and phosphine (aromatic substituents) ligands. The bisdiimine-type ligand has a more extended π-system than aromatic substituents in phosphine ligands. Therefore, the lower energy bands (>270 nm) belong to their LC (ligand-centered) transitions, whereas other bands in the higher energy region correspond to the bisphosphines LC transitions. Complexes **1** and **5** also display shoulders around 315–330 nm, which can be assigned to the electron transitions within the benzene or binaphthyl fragments in the corresponding phosphine. The non-intense broad absorptions also have been observed in the longer wavelength region (>350 nm). These bands are attributed to the MLCT transition from the Cu(I) ion to the diimine ligand [29,30,31,32,33,34,35,36,37].

In the DCE solution, complexes **1**–**4** exhibit unstructured broad bands centered at ca. 575–610 nm (Figure 2b, Table 2). Differences in the emission maxima of **1**–**4** suggest that the phosphine ligand affects the photophysical properties. Complexes **1**–**4** possess decay times at the microsecond domain (8–10 µs) in the deoxygenated DCE solution and its intensity drops to zero upon oxygenation, indicating the phosphorescence or delayed fluorescence [31,40,48]. On the other hand, close values of decay times suggest the absence of significant differences in relaxation pathways such as vibrational. This also suggests the phosphine ligand affects the emission only due to different donation ability, and the complexes’ geometries practically level out in the solution. The observed behavior correlates to the published data on the influence of electronic and steric effects of substituents in diimine ligands [40]. Interestingly, in the case of complex **5,** the emission maximum was observed in the higher-energy region (495 nm). The band observed can be attributed to the emission of charge-transfer nature. The LC phosphorescence bands typical for binaphthyl systems were not observed in the solution [49]. The emission decay of complex **5** demonstrates the nanosecond lifetimes (2 ns) and fluorescent nature of emission. Such behavior could be rationalized by the difference in geometries of excited states of different phosphines (vide infra).

The efficiency of the photoluminescence of complexes **1**–**4** tends to zero (<0.1%) in the deoxygenated DCE solution. Upon oxygenation, the photoluminescence intensity almost drops to zero, indicating the phosphorescence or delayed fluorescence. In contrast, the QY for complex **5**, possessing an ^1^MLCT nature is 1.7% and it is just slightly oxygen-sensitive, which indicates the fluorescent nature of the observed glow.

In the solid-state, complexes **1**–**4** display broad unstructured bands of MLCT nature in the yellow-green region (Figure 2c, Table 2). The temperature decrease leads to the redshift of the emission maxima and significant elongation of the decay times. These data correlate with the typical delayed fluorescence for this type of compound [30,33,36,37,40]. Complex **5** displays typical LC phosphorescence (118 µs) within the binaphthyl ligand.

**Figure 3:**
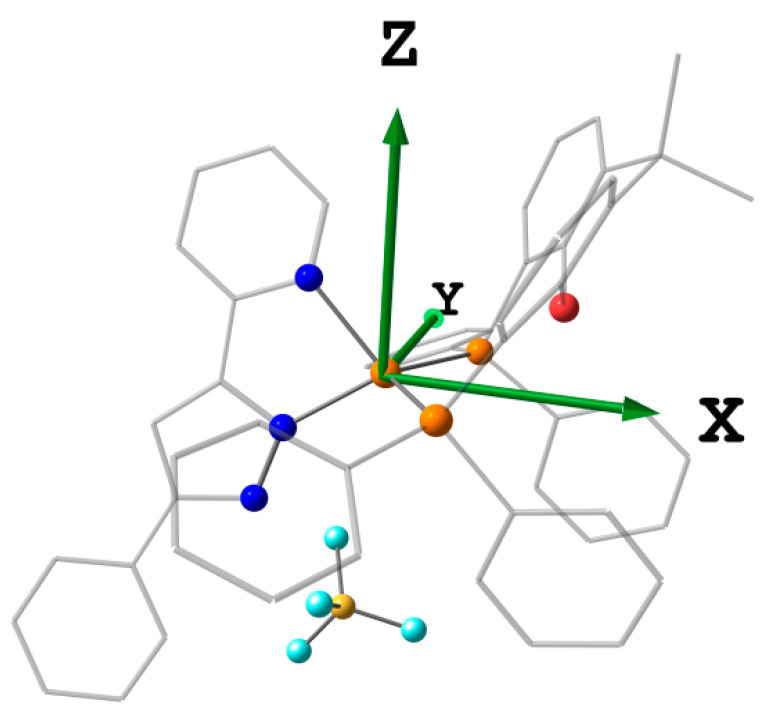
Principal axes used for buried volume steric maps. The Cu atom is the origin of coordinates, the XY plane is the Cu–P–P plane, and the *X*-axis is set to pass through the midpoint of P–P atoms.

Our data show that the efficiency of photoluminescence in the solid-state also significantly depends on the coordinated phosphine. A higher QY has been observed for complex **2** with a bulky and rigid Xantphos ligand (78%). Complexes **1** and **3** demonstrate the approximate efficiency as 0.23 and 0.28, respectively. Complex **4** with PPh_3_ demonstrates expectedly low QY due to structural flexibility. Based on the results obtained, we propose that the photophysical properties, especially photoluminescence QYs, of copper(I) complexes with Pyridine-pyrazoles and phosphines are affected by the steric effects of the ligands and the overall structural rigidity of complex, rather than by the donor ability of ligands. To our surprise, the role of the phosphine ligand has not been studied for the pyridine–pyrazole copper(I) complexes, even though the importance of the steric bulk has been demonstrated for bipyridine-containing analogs [42,43]. To confirm our hypothesis, we analyzed free volume around copper atoms in complexes **1**–**3** and in analogs [^Ph^Pz^Me^PyCu(DPEPhos)]BF_4_ that contain a Me substituent at the Py fragment and demonstrate comparable QY (71%) [40]. The buried volume was calculated by the SAMBVCA 2.1 program [50]. Molecule orientation is depicted in Figure 3.

**Figure 4:**
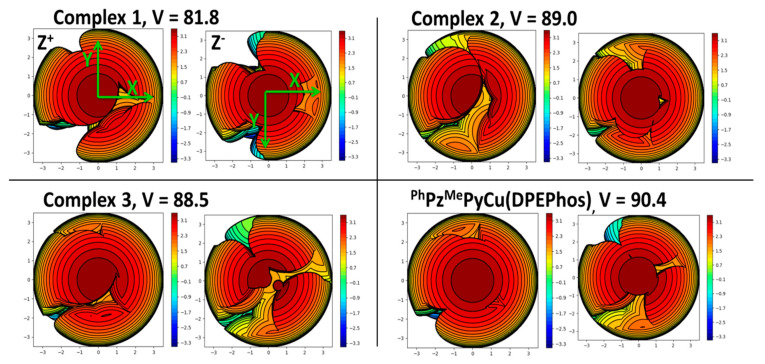
The buried volume representation for complexes **1**–**3** and published ^Ph^Pz^Me^PyCu(DPEPhos) [40], from the top (positive *Z*−axis, pyridine side, left portion) and from the bottom (negative *Z*−axis, pyrazole NH···BF_4_ side, right portion).

Complex **1** with the dppbz ligand has a rather large free volume—18.2%. Switching to the DPEPhos ligand (complex **3**) decreases this value to 11.5%. Complex **2**, with the Xantphos ligand possessing the best efficiency, features an even smaller free volume of 11.0%. In the case of [^Ph^Pz^Me^PyCu(DPEPhos)]BF_4_ published by Lu et al. [40], this parameter is 9.6% (Figure 4). Interestingly, in contrast to dppbz- or DPEPhos-containing complexes, which possess the free volume at the side of the BF_4_- counterion, in complex **2** with the Xantphos ligand, the larger part of the free volume is located near the pyridine part of the diimine ligand.

These data suggest that the steric loading of copper atoms leads to the advantageous stabilization of the excited states and increasing photoluminescence efficiency. The donor ability of ligands mainly influences the emission maxima. This ultimately offers two different pathways for ligand modification—steric loading at the ortho-position of pyridine should bring an additional benefit for Xantphos containing complexes. At the same time, the traditional N-substitution of pyrazole should effectively shield metal atoms.

**Figure 5:**
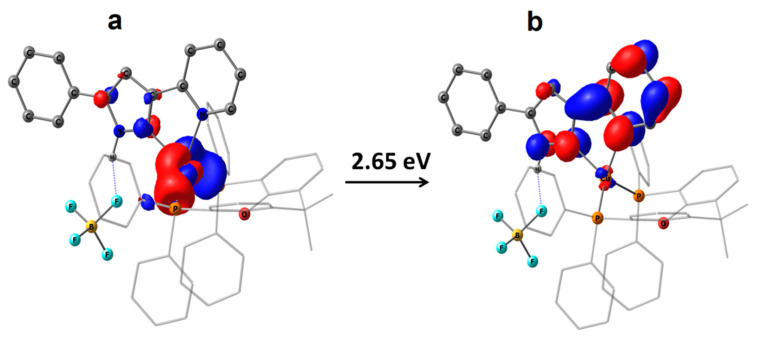
Natural transition orbitals HONTO (**a**) and LUNTO (**b**) for the S_0_→S_1_ excitation of **2** as an isosurface at 0.05 a.u.

DFT calculations shed some light on the observed emission pattern. The optimized geometries of complexes **1**–**3**, **5** (with the BF_4_ counterions) in the ground state qualitatively reproduce tetrahedral X-ray geometry. The τ*_δ_* indices are 0.81 and 0.84 for **2** and **3**, while for **5** (0.75) and especially for **1** (0.65) they are significantly lower, being on the edge of the tetrahedral and sawhorse geometries. The TD-DFT analysis suggests that the lowest energy singlet excitations are rather intensive (f~0.1) MLCT(Cu + P→N^N) transitions for all complexes (Table 3, Figure 5). Thus, for the complexes studied, the geometries of TD-DFT optimized S_1_ states are significantly different from the distorted tetrahedra of the ground state. As it was supposed for Cu(I) excitation [51] it drifts to the square planar geometry, and this is more pronounced for the complex **1**, which features the smallest natural bite angle of diphosphine [23,52,53] (β_n_ = 83°) suitable for the square planar geometry. Notably, for ether-based diphosphines (DPEPhos and Xantphos) with larger bite angles (102 and 108°) [40], the square planar geometry of P^P and N^N ligands was not observed for the geometry of the S_1_ state (Table 4). Instead, they converged to the square-pyramidal one, with BF_4_ counterion occupying a position at the pyramid base and one phosphine moving to the vertex [23,52,53]. The same is true for complex **5** with the BINAP ligand (β_n_ = 92°).

**Figure 6:**
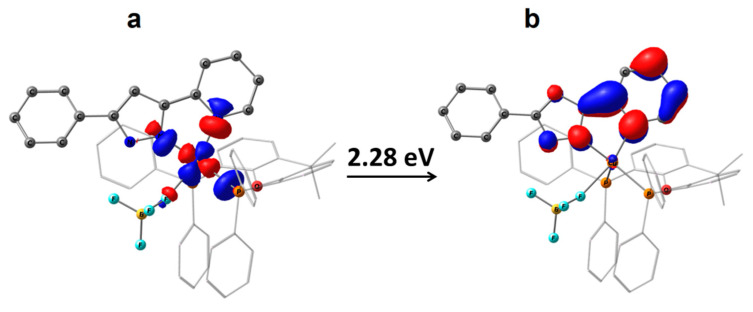
LSOMO (**a**) and HSOMO (**b**) contours for restricted open-shell triplet state T^pyr^ of **2** as isosurface at 0.05 a.u.

**Figure 7:**
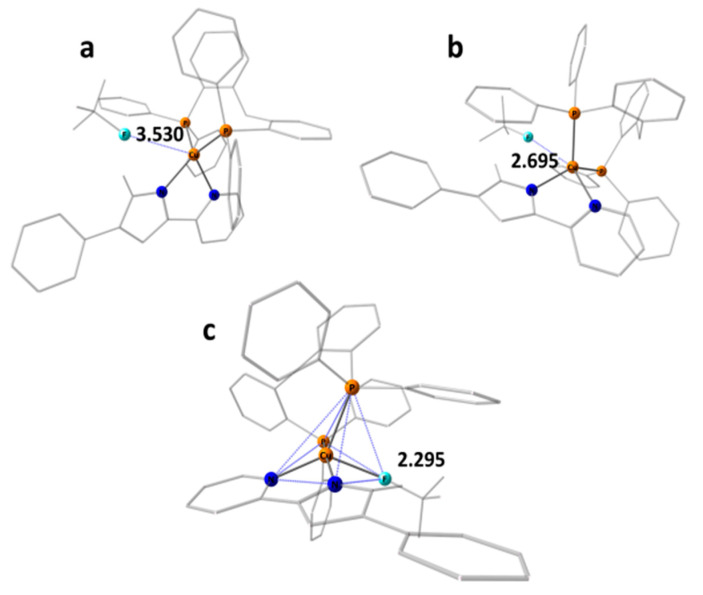
Representations of the tetrahedral ground state (**a**), tetrahedral triplet (**b**), and pyramidal triplet (**c**) for **3**. All atoms except N, P, and F coordinated to copper are shown as a wireframe. Cu–F distances are shown in Å, angles are summarized in Table S2.

The TD-DFT analysis of the spin forbidden S_0_→T_1_ transitions reveals that the ^3^MLCT state complimentary to the ^1^MLCT excitation is among the lowest four triplet states. Moreover, the energy difference between the lowest triplet and MLCT triplet does not exceed 0.4 eV for **1**–**3** but becomes magnificent 0.8 eV for **5**. This suggests that **1**–**3** could successfully interconvert to the ^3^MLCT state even if it is not the lowest, whereas **5** could fall into a very favorable binaphthyl centered ^3^LC state. The triplet state optimizations (in the framework of both unrestricted (UDFT) and restricted-open shell (RODFT) DFT with S = 3) result in the two possible geometries of triplets for **2** and, **3** whereas **1** and **5** demonstrate only one possible geometry. The lowest energy triplet pyramidal geometry (designated as T^pyr^) shows a minimal distortion from the S_1_ geometry for all complexes and its SOMO orbitals correspond to the MLCT (Cu + P→N^N) transitions (Figure 6). The second isomer, designated as T^tetr^, which keeps the tetrahedral surrounding of copper, is unfavored by 0.6–0.9 eV, and was found only for **2** and **3**. Notably, **2-T^pyr^** and **3-T^pyr^** triplets lie lower than the optimized S_1_ state of corresponding complexes. In contrast, **2-T^tetr^** and **3-T^tetr^** triplets are higher by energy and could be populated only by using a higher energy excitation. S_1_→T^pyr^ interconversion, which required minimal geometry changes, would be preferred if the molecule exited to the first singlet. Still, significant geometry changes are required for the molecule to be rested at the S_1_ excited state (Figure 7, Table 5). These changes are smaller for rigid Xantphos-containing complex **2**. We suggest that despite both the lowest excited singlet and triplet for **5** were located as a ^3^MLCT state, and it requires an even smaller geometry distortion to relax the S_1_ state, the ^3^LC emission pathway in a solid is preferred since it does not require any geometry changes. The geometry relaxation is much easier in solution and thus it allows a successful population of the lowest energy triplet state for **5,** changing the emission profile to ^1^MLCT. The PL pathway for **1**–**4** in solids is S_0_→S_1_→T_1_→S_0_ leading to the excitation at 370–410 nm and ^3^MLCT emission around 550 nm. Therefore, QYs could be correlated with the required geometry changes upon S_0_→S_1_ excitation and T_1_→S_0_ phosphorescence: the larger geometry changes of **1**–**4** hamper the effective light emissions. Likewise, the range of geometry changes can be correlated with the steric loading of the metal—a larger buried volume leads to smaller geometry changes in an excited state.

## 3. Materials and Methods

^1^H, ^19^F, and ^31^P NMR measurements were carried out on Avance 400 spectrometer (Bruker). Infrared (IR) spectra were collected on a IRPrestige 21 FT-IR spectrometer (Shimadzu) in nujol. The UV-vis spectra of solutions were measured on Cary 50 Uv-Vis Spectrophotometer (Varian). The diffuse reflectance spectra of solid samples were measured on UV-2600 Uv-Vis Spectrophotometer (Shimadzu) equipped with ISR-2600Plus Integrating Sphere Attachment (Shimadzu). The photoluminescence spectra were recorded at 77K and 298K on the Fluorolog-3 spectrofluorometer system (HORIBA Jobin Yvon S.A.S.) The excitation source was a 450 W Xenon lamp with Czerny–Turner double monochromators; the registration channel was equipped by R928 photomultiplier with Czerny–Turner double monochromators. 150 W pulsed Xenon lamp was used for phosphorescence decay curves measurements. The phosphorescence decay curves were analyzed using the FluoroEssence^TM^ software (HORIBA Jobin Yvon S.A.S.) for the calculation of the phosphorescence lifetime values. Quantum yields of powder photoluminescence and solution photoluminescence were measured by the absolute method. Light of photoluminescence was collected by Quanta-φ F-3029- sphere linked with Fluorolog 3 by Fiber-Optics adaptor FL-3000 (Horiba Jobin Yvon S.A.S). Photoluminescence quantum yields were calculated by FluoroEssence^TM^ software. Fluorescence decay curves were measured with FluoroHub (Horiba Jobin Yvon S.A.S.) with a NanoLED (368 nm, Horiba Jobin Yvon S.A.S) as an impulse light source. Fluorescence decay curves were analyzed by the DAS 6.6 software (Horiba Jobin Yvon S.A.S) for the definition of lifetime value.

### 3.1. Synthesis and Characterization

All reactions were performed under an argon atmosphere using anhydrous solvents or solvents treated with an appropriate drying reagent. The diimine ligand was synthesized as described [44]. Commercially available (Merck) 1,2-Bis(diphenylphosphino)benzene, 4,5-Bis(diphenylphosphino)-9,9-dimethylxanthene, bis[(2-(diphenylphosphanyl)phenyl] ether, triphenylphosphine, and 2,2′-Bis(diphenylphosphino)-1,1′-binaphthalene were used as received.

General procedure for the synthesis of complexes **1**–**4**. The solution of [Cu(CH_3_CN)_4_]BF_4_ (50 mg, 0.159 mmol) and 3-(2-pyridyl)-5-phenyl-pyrazole (35.1 mg, 0.159 mmol) was stirred in acetone (4 mL) for 30 min, and then, the corresponding amount of phosphine was added (0.159 mmol for bisphosphines and 0.318 for PPh_3_). The resulting slightly yellow-green solution was stirred overnight at room temperature. The solvent was evaporated to dryness under reduced pressure, and the residue was dissolved in 0.5 mL CH_2_Cl_2_. Hexane (2 mL) was added to the solution, and the pale green powder was obtained by precipitation at −10 °C. Then, the solvent was removed by decantation, and the crude product was rubbed with a glass rod at 2 mL of Et_2_O. The solvent was evaporated, yielding the titled compound.

Complex **1**. Yield 98.8 mg (76%). ^1^H NMR (CD_2_Cl_2_) δ = 12.51 (s, 1H, N*H*^Pz^), 7.90 (t, 2H, C*H*^Py^), 7.88 s (s, 1H) 7.78–7.24 (m, 22H, C*H*^Ph^); 7.20 (1H, C*H*^Pz^), 7.12 (t, 4H), 7.02 (m, 4H). ^19^F NMR (CD_2_Cl_2_): δ = −151.2 (s, B*F*_4_); ^31^P{^1^H} NMR (CD_2_Cl_2_) δ = −4.68 (br s, 2P). IR (nujol, cm^−1^): ν 3250 (br s, νN*H*^Pz^), 3143 (νCH^Pz^), 3055, 3037 (νCH^Ar^), 1606, 1586, 1570 (δCN^N^N^). Calc. for C_44_H_35_BF_4_CuN_3_P_2_ (%): C, 64.60; H, 4.31; N, 5.14; Found (%): C, 64.30; H, 4.50; N, 5.01;

Complex **2**. Yield 132.7 mg (88%). ^1^H NMR (CD_2_Cl_2_) δ = 12.11 (s, 1H, NH^Pz^), 7.91 (t, 2H, C*H* ^Py^), 7.71 (t, 4H), 7.45 (t, 4H), 7.39–7.05 (m, 20H, Ph), 6.92 (t, 2H), 7.82 (t, 2H), 6.57 (d, 2H). ^19^F NMR (CD_2_Cl_2_): δ = −151.14 (s, B*F*_4_); ^31^P{^1^H} NMR (CD_2_Cl_2_) δ = −13.27 (br s, 2P). IR (nujol, cm^−1^): ν 3255 br s, νN*H*^Pz^), 3145 (νCH^Pz^), 3056, 3037 (νCH^Ar^), 1604, 1586, 1570 (δCN^N^N^). Calc. for C_53_H_43_BF_4_CuN_3_OP_2_ (%):C, 66.99; H, 4.56; N, 4.42; Found (%): C, 66.89; H, 4.74; N, 4.44; C, 67.01; H, 4.61; N, 4.69; Found (%): C, 67.15; H, 4.50; N, 5.00;

Complex **3**. Yield 125.67 mg (87%). ^1^H NMR (CD_2_Cl_2_) δ = 12.61 (s, 1H, NH^Pz^), 7.97 (s, 1H,), 7.89 (t, 2H^Py^), 7.73 (d, 2H), 7.61–7.11 (m, 22 H), 7.07 (d, 4H) 6.98 (t, 2H), 6.89 (m, 3H), 6.74 (t, 2H). ^19^F NMR (CD_2_Cl_2_): δ = −151.03 (s, B*F*_4_); ^31^P{^1^H} NMR (CD_2_Cl_2_) δ = −12.61 (s, 2P). IR (nujol, cm^−1^): ν 3305 (br s, νN*H*^Pz^), 3143 (νCH^Pz^), 3055, 3051 (νCH^Ar^), 1605, 1588, 1570 (δCN^N^N^). Calc. for C_50_H_39_BF_4_CuN_3_OP_2_ (%): C, 65.98; H, 4.32; N, 4.62; Found (%):C, 65.90; H, 4.45; N, 4.48;

Complex **4**. Yield 115.1 mg (81%). ^1^H NMR (CD_2_Cl_2_) δ = 12.98 (s, 1H, NH^Pz^), 7.88 (t, 2H, C*H* ^Py^), 7.79 (d, 1H), 7.70 (d, 2H), 7.58 (t, 2H), 7.52 (m, 2H), 7.45–7.31 (m, 10H, Ph), 7.45–7.31 (m, 10H, Ph), 7.31–7.16 (m, 20H, Ph), 7.1 (s, 1H); ^19^F NMR (CD_2_Cl_2_): δ = −151.33 (s, B*F*_4_); ^31^P{^1^H} NMR (CD_2_Cl_2_) δ = 1.78 (s, 2P). IR (nujol, cm^−1^): ν 3265 (br s, νN*H*^Pz^), 3142 (νCH^Pz^), 3054, 3039 (νCH^Ar^), 1604, 1587, 1570 (δCN^N^N^). Calc. for C_50_H_41_BF_4_CuN_3_P_2_ (%): C, 67.01; H, 4.61; N, 4.69; Found (%): C, 66.79; H, 4.85; N, 4.49;

Complex **5**. The solution of [Cu(CH_3_CN)_4_]BF_4_ (50 mg, 0.159 mmol) and 3-(2′-pyridyl)-5-phenyl-pyrazole (35.1 mg, 0.159 mmol) was stirred in acetone (4 mL) for 30 min, then BINAP (98.8 mg, 0.159 mmol) was added. The reaction mixture was stirred overnight at room temperature. The solid obtained was filtered off, washed with acetone (3 mL) and hexane (5 mL). The obtained powder was dried under vacuum, yielding 143.6 mg of complex **5** (91%). ^1^H NMR (CD_3_CN) δ = 12.51 (br s, 1H, NH^Pz^), 8.10 (m, 2H, C*H* ^Py^), 7.89 (t, 2H), 7.74 (d, 2H), 7.64 (t, 2H), 7.61–7.20 (m, 20H, Ph), 7.20–6.9 (m, 6H), 6.85 (d, 2H), 6.77 (d, 2H), 6.61 (m, 4H); ^19^F NMR (CD_3_CN): δ = −151.73 (s, B*F*_4_); ^31^P{^1^H} NMR (CD_3_CN) δ = 0.68 (s, 2P). IR (nujol, cm^−1^): ν 3250 (br s, νN*H*^Pz^), 3146 (νCH^Pz^), 3054, 3036, (νCH^Ar^), 1604, 1586, 1574 (δCN^N^N^). Calc. for C_58_H_43_BF_4_CuN_3_P_2_ (%): C, 70.06; H, 4.36; N, 4.23; Found (%): C, 69.27; H, 4.75; N, 4.04.

### 3.2. Computational Details

Calculations were performed with the dispersion corrected ωB97XD functional [54] applying the def2-SVP basis set [55] for all atoms using Gaussian16 software [56]. Geometry optimizations of the BF_4_^-^ salts **1**–**3** and **5** were made for the ground state without any restrictions. The TDDFT analysis of vertical excitation was performed at the same computational level for the first 50 states. The analysis of natural transition orbitals was performed with the Multiwfn 3.7 program [57]. The optimization of the first excited singlet state S_1_ was performed in the framework of TDDFT. The triplet states geometry optimizations were performed in the framework of conventional DFT applying both unrestricted (UDFT, all electrons unpaired) and restricted open-shell (RODFT, only two unpaired electrons) leading to essentially the same geometry with both approaches. The analysis of SOMO orbitals was performed for the RODFT wavefunctions.

The buried volume was calculated by the SAMBVCA 2.1 program [50]. Values of calculation parameters have been used as defaulted by the program: atomic radii are Bondi radii multiplied by 1.17 [58]. The radius of the integration sphere was 3.5 A; hydrogen atoms are not included in the calculation.

### 3.3. Crystal Structure Determination

The structures were solved by direct method and refined in anisotropic approximation for non-hydrogen atoms. Hydrogen atoms of methyl and aromatic fragments were calculated according to those idealized geometries and refined with constraints applied to C–H and N–H bond lengths and equivalent displacement parameters (U_eq_(H) = 1.5U_eq_(Y), Y—central atom of YH₃ group). Analysis of residual electron density and displacement parameters in **1** and **3** has shown that solvent molecules and BF_4_ anions are disordered. All structures were solved with the ShelXT [59] program and refined with the ShelXL [60] program. Molecular graphics were drawn using OLEX2 [61] program. The supplementary crystallographic data for complexes **1**–**3**, and **5** can be found in CCDC 2111465 – 2111468 files. These data can be obtained free of charge from the Cambridge Crystallographic Data Center via https://www.ccdc.cam.ac.uk/structures. Crystal data and structure refinement parameters are presented in Table S1.

## 4. Conclusions

Emissive Cu(I) cationic complexes with 3-(2-pyridyl)-5-phenyl-pyrazole and various phosphines were designed and characterized. The compounds obtained exhibit structural stability in terms of the central copper atom environment. The phosphine ligand’s bite angle determines the copper surrounding in experimental XRD and calculated structures closer to ideal tetrahedral geometry for ether-based diphosphines (τ*_δ_* ca. 0.8–0.9). At that, the properties of phosphines have a dramatic effect on the complexes’ photoluminescence efficiency in the solid-state that varies from close to zero to a notable 78%. Our study has shown that, that for high QY of MLCT phosphorescence, one requires bulk loading of the metal atom, which could be effectively achieved by changing phosphine from DPEPhos to Xantphos. Notably, this type of change is rare in copper(I) pyrazolates photochemistry since most researchers prefer synthetic modifications in the pyrazole-pyridine unit while using a simple DPEPhos ligand. Although diimine modification still allows effective light emission, the DPEPhos-Xantphos change brings not only QY gain but also changes a steric map around the Cu atom. Deprotonation of pyrazole in such complexes allows obtaining neutral complexes. This process should be less painful for a bulky environment of the metal atom as, e.g., in the case of the Xantphos ligand since it provides more effective shielding of hemisphere containing the NH···BF_4_ moiety. In addition, traditional *o*-substitution in pyridine fragments can provide additional steric loading. All this opens a promising possibility for the synthesis of neutral PyPz-P^P copper(I) complexes, which should be more convenient for the development of OLED devices. The Xantphos ligand provides not only steric loading but also requires smaller geometry changes upon excitation to S_1_ and upon emission from T_1_ to the ground state.

Interestingly, complex **5** containing the BINAP ligand requires fewer geometry changes for these transitions. This complex includes an extended aromatic binaphthyl system being very potent for ligand-centered emission, which eventually does not require any significant geometry distortion. In contrast to **1**–**4**, this complex exhibits fluorescence in the DCE solution due to facile relaxation of the singlet excited state, changing the emission profile to ^1^MLCT.

## Figures and Tables

**Table 1:** Selected structural parameters and τ*_δ_* values for **1**–**3**, **5** and reported analogs.

	Largest Angle, α (Deg)	Second-Largest Angle, *β* (Deg)	τ_δ_	References
**1**	130.89	128.78	0.71	this work
**2**	123.21	121.61	0.81	this work
**3**	119.75	117.48	0.85	this work
**5**	128.93	127.18	0.73	this work
^Ph^Pz^Me^PyCu(DPEPhos)	120.65	117.16	0.84	[40]
^F^Pz^Me^PyCu(DPEPhos) ^1^	120.0	118.92	0.85	[35]
^F^Pz^Me^PyCu(DPEPhos) ^2^	119.73	119.05	0.85	[35]
^Ph^TrzPy ^a^ Cu(DPEPhos) ^a^	117.1	116.8	0.89	[33]
^t^BuTrzPyCu(dppf) ^b^	123.88	120.96	0.79	[38]
^F^TrzPyCu(dppf)	126.58	114.99	0.76	[38]

^1,2^: one of two possible isomers; ^a^: Trz = triazole; ^b^: dppf = 1,1′-bis(diphenylphosphino)ferrocene.

**Table 2:** Photophysical parameters for complexes **1**–**5**.

	Solution		Solid				
	λ_max_ (ε∙10^−4^, M^−1^ cm^−1^), nm	λ_max_^em^ (λ^ex^), nm	λ_max_^em^ (λ^ex^), nm; 298 K	τ^298^, µs	λ_max_^em^ (λ^ex^), nm; 77 K	τ^77^, µs	Φ^298,^ %
**1**	287 (2.86); 315 (1.33); 378 (0.59)	574 (420)	545 (420)	19.4 ± 0.2	585 (410)	404 ± 4	23
**2**	278 (3.43); 349 (0.35)	580 (385)	522 (370)	66.2 ± 0.6	561 (370)	252 ± 2	78
**3**	280 (2.8); 353 (0.41)	605 (385)	559 (370)	22.7 ± 0.1	610 (370)	186 ± 1	28
**4**	267 (3.6); 340 (0.44)	610 (385)	540 (370)	28.9 ± 0.5	548 (370)	132 ± 1	5
**5**	287 (2.73); 328 (0.78); 373 (0.60)	495 (430)	LC bands ^a^	118 ± 1	LC bands ^a^	4456 ± 34	1

^a^ LC bands of **5** at RT (ex. 420 nm): 501, 538, 576, 642 at 77 K (ex. 410 nm): 502, 518, 532, 557, 579, 596, 659.

**Table 3:** DFT/TDDFT calculated energies of different electronic states, relative to the S_0_ in eV.

	S_1_	UDFT T^pyr a^	RODFT T^pyr b^	UDFT T^tetr^	RODFT T^tetr^
**1**	2.61	2.23	2.34	-	-
**2**	2.65	2.28	2.38	3.14	3.29
**3**	2.88	2.47	2.57	3.01	3.16
**5**	2.91	2.47	2.57	-	-

^a^ unrestricted DFT ^b^ restricted-open shell DFT.

**Table 4:** P–M–P angles in X-ray and DFT structures.

	X-Ray	DFT (S_0_)	TDDFT (S_1_)	DFT (T^pyr^)
**1**	92.07(4)	89	80	80
**2**	112.03(3)	114	101	103
**3**	115.08(3)	114	97	99
**5**	101.65(3)	100	92	92

**Table 5:** Geometry differences between different electronic states as a sum of copper angle differences (modulo).

	S_0_/S_1_	S_0_/T^pyr^	S_0_/T^tetr^	S_1_/T^pyr^
**1**	135	133	-	5
**2**	91	80	20	13
**3**	107	100	44	11
**5**	74	73	-	11

## Data Availability

The data presented in this study are available in article and supplementary materials.

## Supplementary Materials

The following are available online, Table S1: Crystal data, data collection, and structure refinement parameters for **1**–**3** and **5**.; Figure S1: Phosphorescence decays of complexes **1**–**5** in the solid state at 298 and 77 K.; Figure S2: Phosphorescence decays of complexes **1**–**4** in deoxygenated DCE solution at 298 K.; Figure S3: Fluorescence decay of complex **5** in the DCE solution at 298 K. Brown—sample, blue–prompt.; Table S2: Angles of DFT and TDDFT optimized complexes.; Table S3: The hole-electron analysis of 10 lowest S_0_-S_x_ transitions. The impacts of fragments to the ground and excited states are shown in percentage.; Figure S4: Natural transition orbitals (HONTO, left and LUNTO, right) for S_0_→S_1_ transition of complexes **1**–**3**, **5** as isosurfaces at 0.05 a.u. Hydrogen atoms are removed; C, O atoms of P^P ligands are shown as a wireframe.; Figure S5: HSOMO (left) a HSOMO-1 (right) for restricted open-shell triplet state T^pyr^ of **1**–**3**, **5** as isosurface at 0.05 a.u. Hydrogen atoms are removed; C, O atoms of P^P ligands are shown as a wireframe.; Figure S6: UV-vis spectra of complexes **1**–**5** in the solid state.; Table S4. The hole-electron analysis of 4 lowest spin forbidden S_0_**→**T_x_ transitions. The impacts of fragments to the ground and excited states are shown in percentages.; Figure S7: Graphical representation of energies of TDDFT computed spin forbidden S_0_→T_x_ transitions for **1**–**3**, **5**. Black—MLCT states, red—P^P ligand centered states, blue N^N ligand centered states. Since the excitation pathway is of MLCT nature, the MLCT triplet state would be preferred until the alternative state becomes extremely profitable.; Figure S8: ^1^H NMR spectrum of complex **1** in CD_2_Cl_2_, inset ^19^F spectrum.; Figure S9: ^31^P{^1^H} NMR spectrum of complex **1** in CD_2_Cl_2_.; Figure S10: ^1^H NMR spectrum of complex **2** in CD_2_Cl_2_, inset ^19^F spectrum**.;** Figure S11: ^31^P{^1^H} NMR spectrum of complex **2** in CD_2_Cl_2_; Figure S12. ^1^H NMR spectrum of complex **3** in CD_2_Cl_2_, inset ^19^F spectrum.; Figure S13: ^31^P{^1^H} NMR spectrum of complex **3** in CD_2_Cl_2_.; Figure S14: ^1^H NMR spectrum of complex **4** in CD_2_Cl_2_, inset ^19^F spectrum.; Figure S15: ^31^P{^1^H} NMR spectrum of complex **4** in CD_2_Cl_2_.; Figure S16: ^1^H NMR spectrum of complex **5** in CD_3_CN, inset ^19^F spectrum Figure S17: ^31^P{^1^H} NMR spectrum of complex **4** in CD_2_Cl_2_. Figure S18: IR spectra of complexes in nujol.

## Sample Availability

Samples of the compounds are not available from the authors.
