# Supplementary material for: Mononuclear Copper(I) 3-(2-pyridyl)pyrazole Complexes: The Crucial Role of Phosphine on Photoluminescence
Source: Molecules. 2021 Nov 14;26(22):6869. doi: 10.3390/molecules26226869 (PMC8620892; doi:10.3390/molecules26226869)
Supplement: Supplementary file 1 [file molecules-26-06869-s001.zip › molecules-1463132-supplementary.pdf]

Supporting Information for

Mononuclear copper(I) 3-(2-pyridyl)pyrazole complexes: the crucial role of phosphine on photoluminescence

Kristina F. Baranova [1,2], Aleksei A. Titov [1]*, Alexander F. Smol'yakov [1,3], Andrey Yu. Chernyadyev [4], Oleg A. Filippov [1]* and Elena S. Shubina[1]

[1] A. N. Nesmeyanov Institute of Organoelement Compounds, Russian Academy of Sciences, Vavilov Str., 28,

119991 Moscow, Russia; krisbar99@gmail.com (K.F.B.); tit@ineos.ac.ru (A.A.T.); h-bond@ineos.ac.ru (O.A.F.);

rengenhik@gmail.com (A.F.S.), shu@ineos.ac.ru (E.S.S.);

[2] Faculty of Chemistry, Lomonosov Moscow State University, 1-3 Leninskie Gory, 119991 Moscow, Russia krisbar99@gmail.com (K.F.B.);

[3] Plekhanov Russian University of Economics, Stremyanny per. 36, Moscow, 117997, Russia; rengenhik@gmail.com (A.F.S.)

[4] A. N. Frumkin Institute of Physical Chemistry and Electrochemistry, Russian Academy of Sciences,

Leninsky prosp. 31/4, 199071 Moscow, Russia; chernyadyev@mail.ru (A. Y. C.);

***** Correspondence: tit@ineos.ac.ru (A.A.T.); h-bond@ineos.ac.ru (O.A.F.); Tel.: +7-499-135-1871

Table S1. Crystal data, data collection, and structure refinement parameters for **1-3** and **5**.

Complex	1	2	3	5
Empirical formula	$C_{44.5}H_{36}BClCuF_4N_3P_2$	$C_{53}H_{43}BCuF_4N_3OP_2$	$C_{50}H_{39}BCuF_4N_3OP_2$	$C_{58}H_{43}BCuF_4N_3P_2$
Formula weight	860.50	950.19	910.13	994.24
Temperature/K	120	120	120	100
Crystal system	triclinic	monoclinic	monoclinic	triclinic
Space group	*P*-1	$P2_1/n$	$P2_1/c$	*P*-1
a/Å	10.511(2)	9.8935(5)	12.724(12)	11.9704(3)
b/Å	18.182(4)	22.2115(11)	18.596(16)	13.4472(3)
c/Å	24.983(5)	20.8288(11)	21.213(17)	15.8231(3)
α/°	100.721(5)	90	90	91.1990(10)
β/°	101.129(5)	102.5590(10)	99.78(2)	97.1030(10)
γ/°	106.111(5)	90	90	102.3720(10)
V/Å^3	4353.1(15)	4467.6(4)	4946(7)	2465.82(10)
Z	4	4	4	2
ρ_{calc}g/cm^3	1.313	1.413	1.222	1.339
μ/mm^{-1}	0.688	0.621	0.558	0.565
F(000)	1764.0	1960.0	1872.0	1024.0
Crystal size/mm^3	0.38 × 0.25 × 0.03	0.24 × 0.08 × 0.01	0.24 × 0.18 × 0.1	0.24 × 0.16 × 0.12
Radiation	MoKα (λ = 0.71073)	MoKα (λ = 0.71073)	MoKα (λ = 0.71073)	MoKα (λ = 0.71073)
2Θ range for data collection/°	2.58 to 52	4.006 to 51.998	2.932 to 59.358	4.144 to 51.998
Reflections collected	52861	43794	60866	25932
Independent reflections	17075 [R_{int} = 0.0832, R_{sigma} = 0.1232]	8785 [R_{int} = 0.0663, R_{sigma} = 0.0484]	13930 [R_{int} = 0.0849, R_{sigma} = 0.1246]	9672 [R_{int} = 0.0369, R_{sigma} = 0.0472]
Data/restraints/parameters	17075/81/1034	8785/0/588	13930/0/586	9672/0/626
Goodness-of-fit on F^2	1.046	1.014	1.088	1.024
Final *R* indexes [I>=2σ (I)]	R_1 = 0.0590, wR_2 = 0.1412	R_1 = 0.0417, wR_2 = 0.0940	R_1 = 0.0543, wR_2 = 0.1439	R_1 = 0.0432, wR_2 = 0.1086
Final *R* indexes [all data]	R_1 = 0.0976, wR_2 = 0.1574	R_1 = 0.0652, wR_2 = 0.1077	R_1 = 0.0960, wR_2 = 0.1579	R_1 = 0.0538, wR_2 = 0.1150
Largest diff. peak/hole / e Å^{-3}	1.42/-0.59	0.69/-0.28	0.71/-0.43	0.71/-0.43

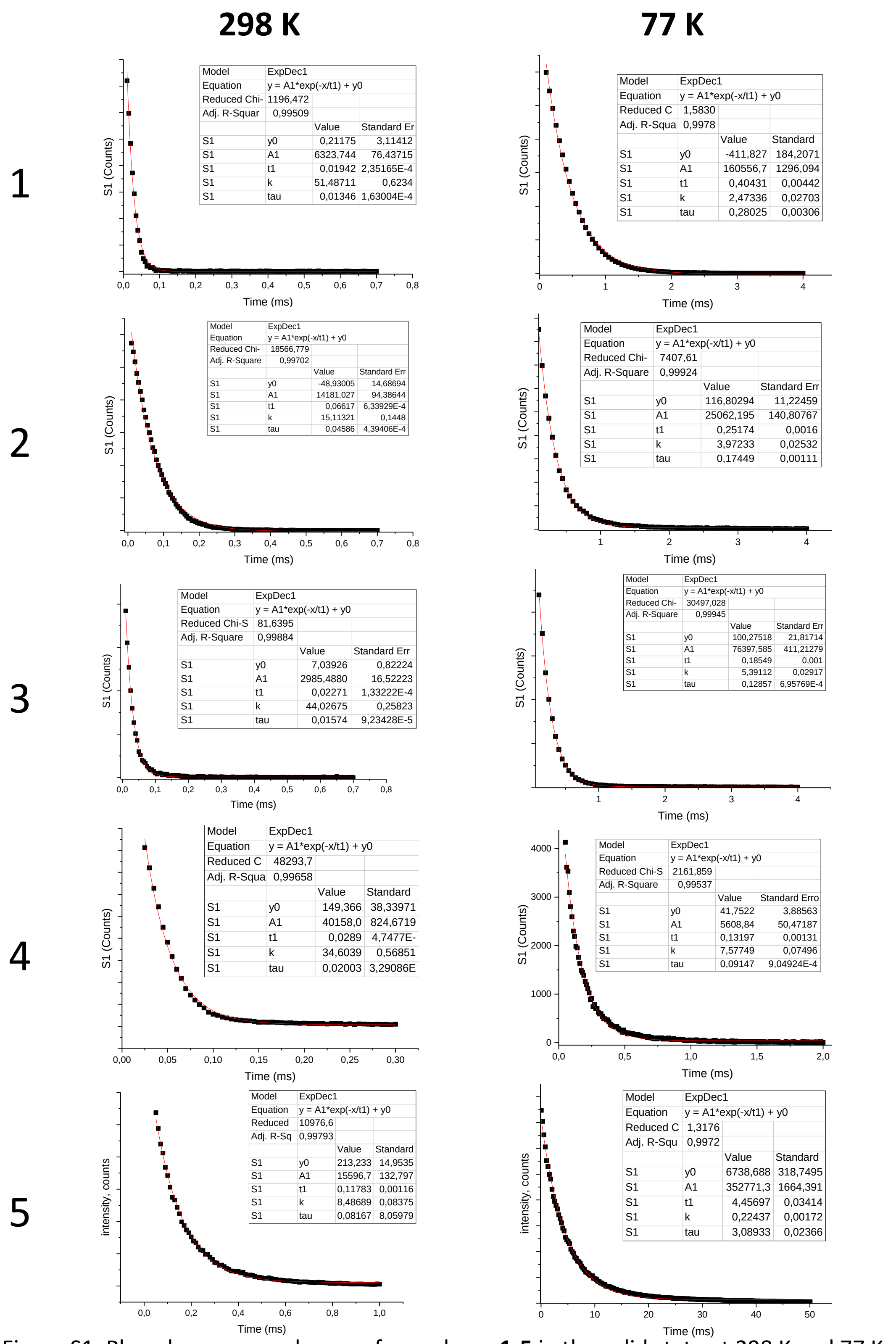

Figure S1. Phosphorescence decays of complexes **1-5** in the solid state at 298 K and 77 K.

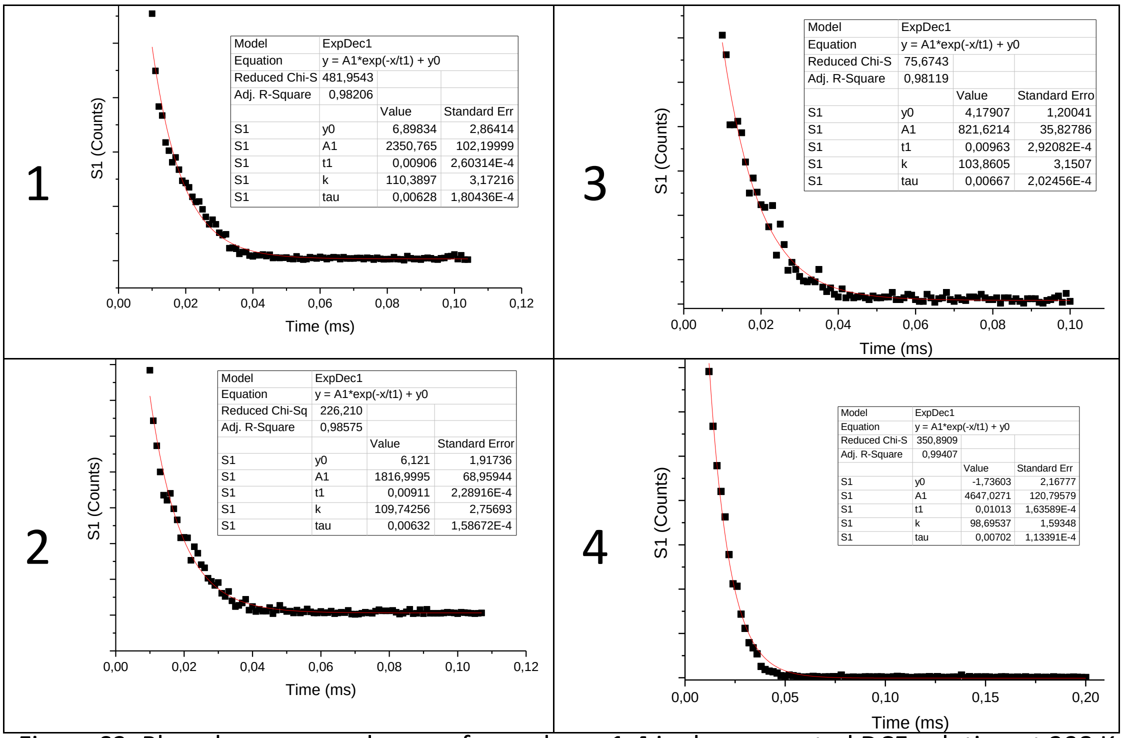

Figure S2. Phosphorescence decays of complexes **1-4** in deoxygenated DCE solution at 298 K.

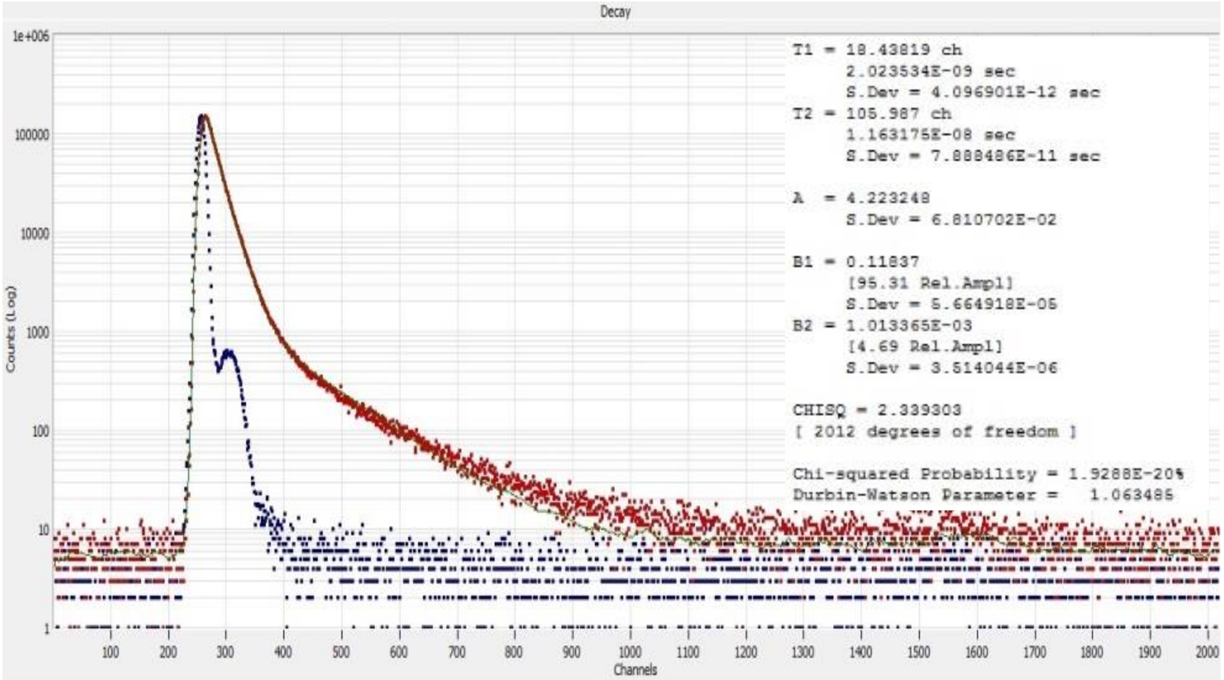

Figure S3. Fluorescence decay of complex **5** in the DCE solution at 298 K. Brown – sample, blue –prompt.

Table S2. Angles of DFT and TDDFT optimized complexes.

	3				2				5			1		
	GS	T^{tetr}	T^{Pyr}	S_1	GS	T^{tetr}	T^{Pyr}	S_1	GS	T^{Pyr}	S_1	GS	T^{Pyr}	S_1
P-Cu-P	114	113	99	97	114	112	103	101	100	92	92	89	80	80
N-Cu-N	77	77	80	80	77	76	81	81	76	82	81	78	80	80
N^{Pz}-Cu-P1	117	104	91	89	118	115	97	95	126	104	103	133	93	93
N^{Pz}-Cu-P2	123	141	166	171	121	129	158	163	127	156	161	134	168	167
N^{Py}-Cu-P1	114	105	127	125	111	108	112	108	124	130	126	108	149	151
N^{Py}-Cu-P2	102	104	101	101	108	105	102	102	99	101	101	107	101	101

Table S3. The hole-electron analysis of 10 lowest S_0-S_x transitions. The impacts of fragments to the ground and excited states are shown in percents.

1

				Ground state					Excited state:				
state	E, eV	λ, nm	f	Cu	P2	ligand	PhPz	BF4	Cu	P2	ligand	PhPz	BF4
S1	3.752	330	0.102	50	26	13	11	0	2	1	4	93	0
S2	3.873	320	0.001	63	4	2	31	1	2	1	2	95	0
S3	4.098	303	0.005	41	34	20	4	0	6	26	66	2	0
S4	4.184	296	0.016	35	39	22	4	0	0	14	84	2	0
S5	4.566	272	0.009	46	28	15	10	0	0	1	8	91	0
S6	4.694	264	0.192	43	29	21	7	0	5	26	55	14	0
S7	4.748	261	0.059	53	11	8	27	1	1	7	22	70	0
S8	4.752	261	0.093	50	20	16	13	1	3	15	58	24	0
S9	4.793	259	0.012	79	6	6	9	0	2	2	4	93	0
S10	4.798	258	0.018	33	34	29	4	0	1	2	96	1	0

2

				Ground state					Excited state:				
state	E, eV	λ, nm	f	Cu	P2	ligand	PhPz	BF4	Cu	P2	ligand	PhPz	BF4
S1	3.872	320	0.109	50	23	12	15	0	2	1	1	96	0
S2	3.961	313	0.002	65	2	2	30	0	2	1	1	96	0
S3	4.333	286	0.006	72	10	9	9	0	2	1	1	96	0
S4	4.555	272	0.003	37	31	28	5	0	5	19	73	2	0
S5	4.632	268	0.009	45	22	13	20	0	0	0	2	97	0
S6	4.765	260	0.057	33	27	26	14	0	1	13	68	17	0
S7	4.813	258	0.003	55	3	3	38	0	0	0	1	99	0
S8	4.843	256	0.118	43	9	8	40	0	2	3	15	81	0
S9	4.991	248	0.029	30	11	48	11	0	1	6	66	27	0
S10	5.007	248	0.007	71	3	22	3	0	2	2	26	70	0

3

				Ground state					Excited state:				
state	E, eV	λ, nm	f	Cu	P2	ligand	PhPz	BF4	Cu	P2	ligand	PhPz	BF4
S1	3.606	344	0.092	53	19	10	18	0	2	1	1	95	0
S2	4.073	304	0.026	65	9	7	20	0	2	1	1	95	0
S3	4.314	287	0.008	71	11	9	9	0	2	1	3	94	0
S4	4.341	286	0.001	52	20	13	15	0	1	3	11	84	0
S5	4.517	275	0.025	40	28	23	8	0	4	18	61	17	0
S6	4.730	262	0.105	31	30	34	5	0	1	13	85	1	0
S7	4.804	258	0.002	60	5	5	29	0	2	3	10	85	0
S8	4.874	254	0.156	40	27	26	7	0	1	15	69	16	0
S9	4.907	253	0.003	64	7	6	23	0	1	1	2	96	0

S10	4.965	250	0.003	91	2	2	5	0	2	1	1	95	0

5

				Ground state					Excited state:				
state	E, eV	λ, nm	f	Cu	P2	ligand	PhPz	BF4	Cu	P2	ligand	PhPz	BF4
S1	3.696	335	0.138	54	24	9	13	0	2	1	4	93	0
S2	3.958	313	0.013	65	5	4	25	1	2	1	6	91	0
S3	4.067	305	0.026	39	30	26	5	0	2	10	75	13	0
S4	4.241	292	0.052	19	8	70	2	0	2	5	83	10	0
S5	4.397	282	0.018	28	17	50	5	0	1	3	58	38	0
S6	4.422	280	0.009	66	9	18	7	0	2	2	11	86	0
S7	4.432	280	0.013	35	17	41	7	0	1	2	48	49	0
S8	4.503	275	0.004	4	3	92	1	0	1	3	94	2	0
S9	4.541	273	0.007	12	8	79	1	0	1	3	96	1	0
S10	4.619	268	0.015	23	15	57	4	0	1	7	85	6	0

Cu – copper atom, P2 – two P atoms, ligand – organic pert of P^P ligand, PhPz – N^N ligand, BF4 – counterion. Color codes – green >90%, blue 30-90%

1	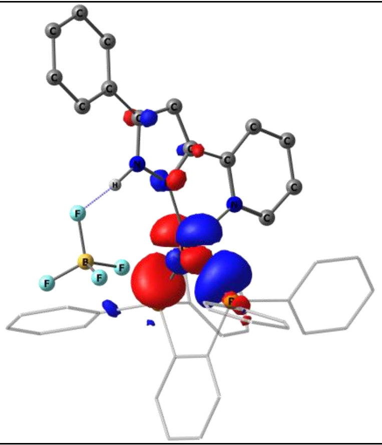	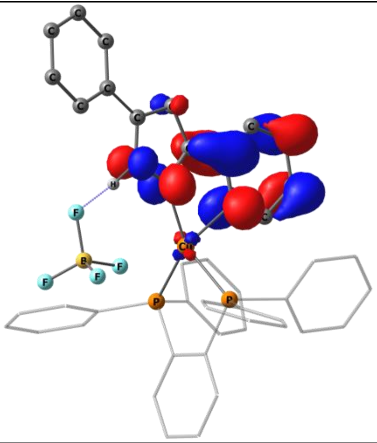
2	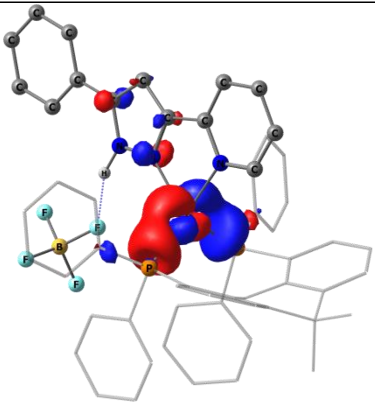	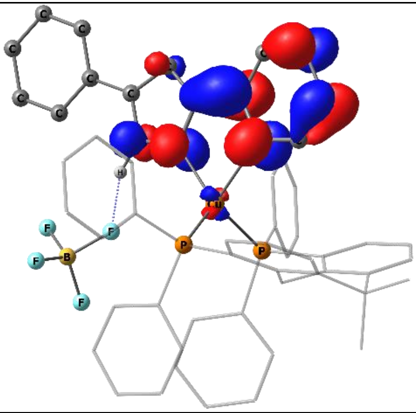
3	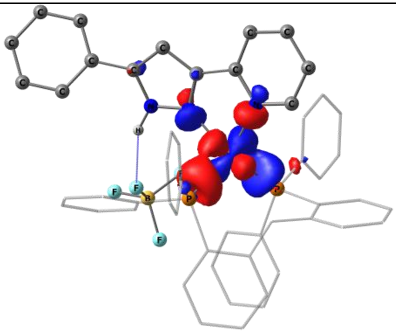	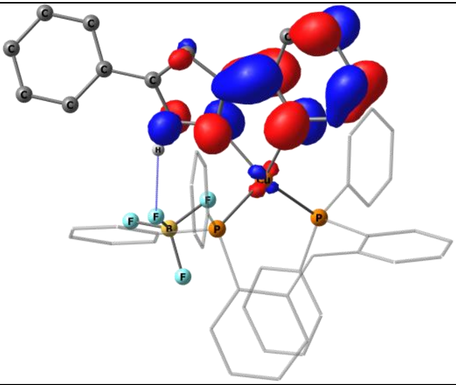
5	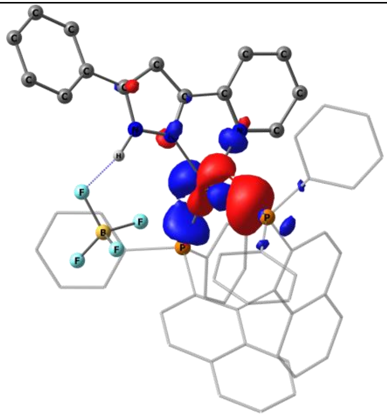	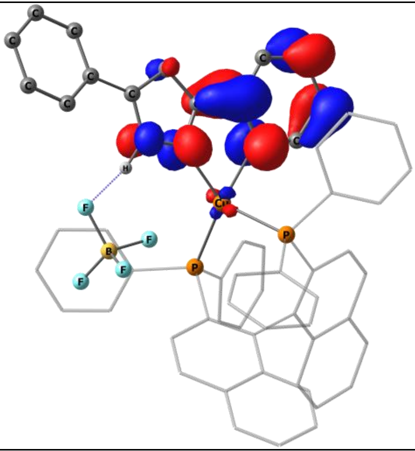

Figure S4. Natural transition orbitals (HONTO, left and LUNTO, right) for $S_0 \rightarrow S_1$ transition of complexes **1-3**, **5** as isosurfaces at 0.05 a.u. Hydrogen atoms are removed; C, O atoms of P^P ligands are shown as a wireframe.

1	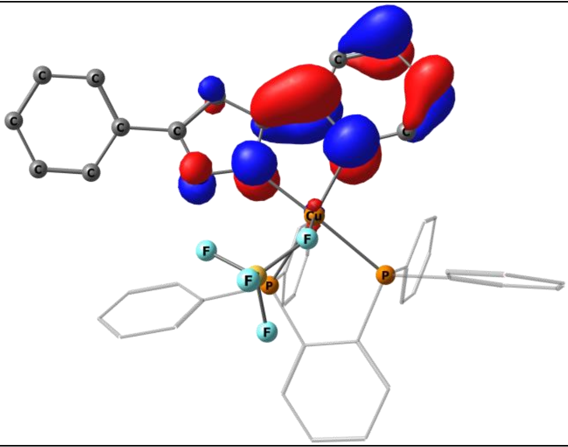	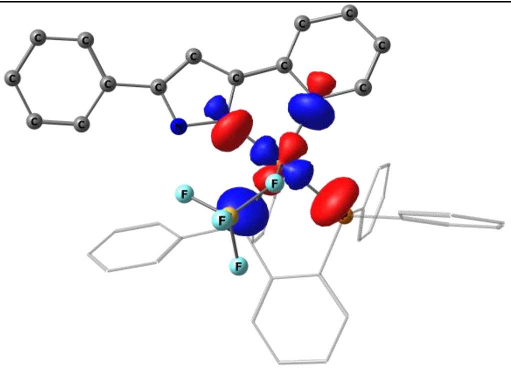
2	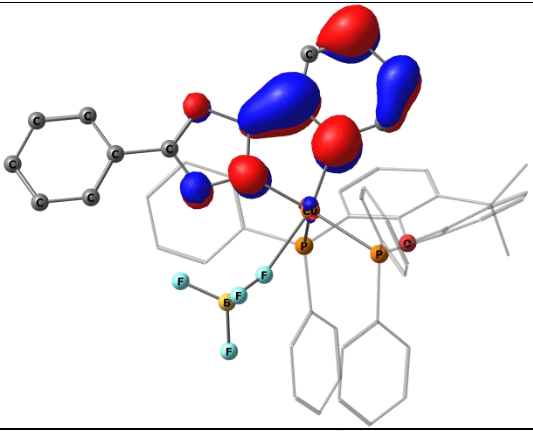	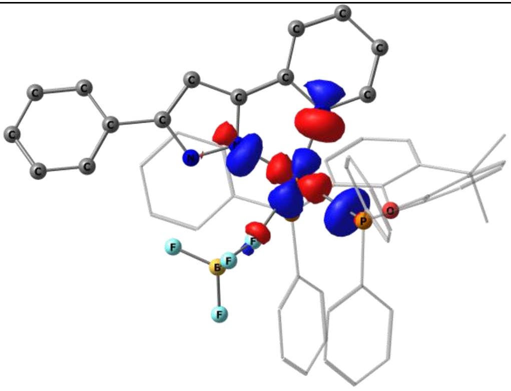
3	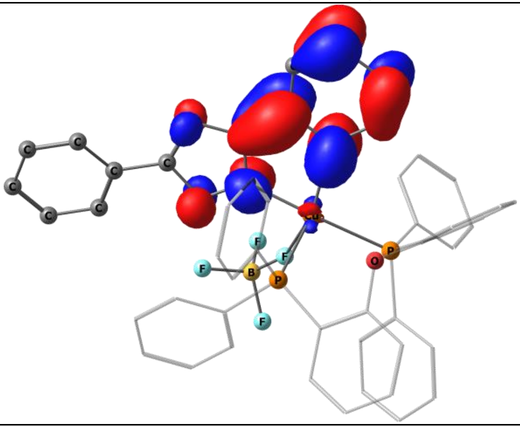	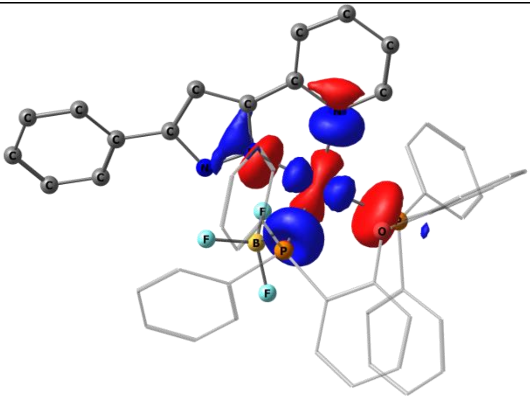
5	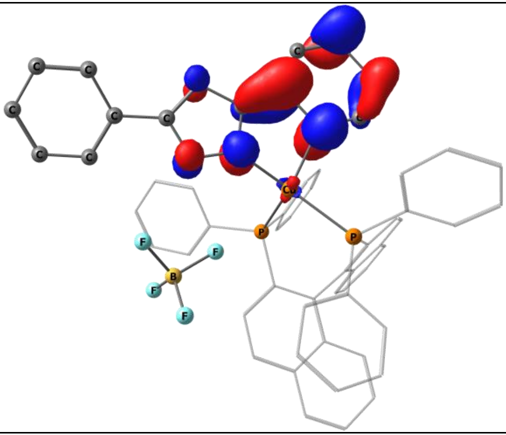	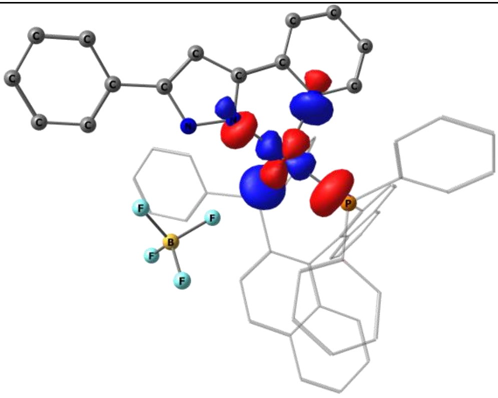

Figure S5. HSOMO (left) a HSOMO-1 (right) for restricted open-shell triplet state T^{pyr} of **1-3**, **5** as isosurface at 0.05 a.u. Hydrogen atoms are removed; C, O atoms of P^P ligands are shown as a wireframe.

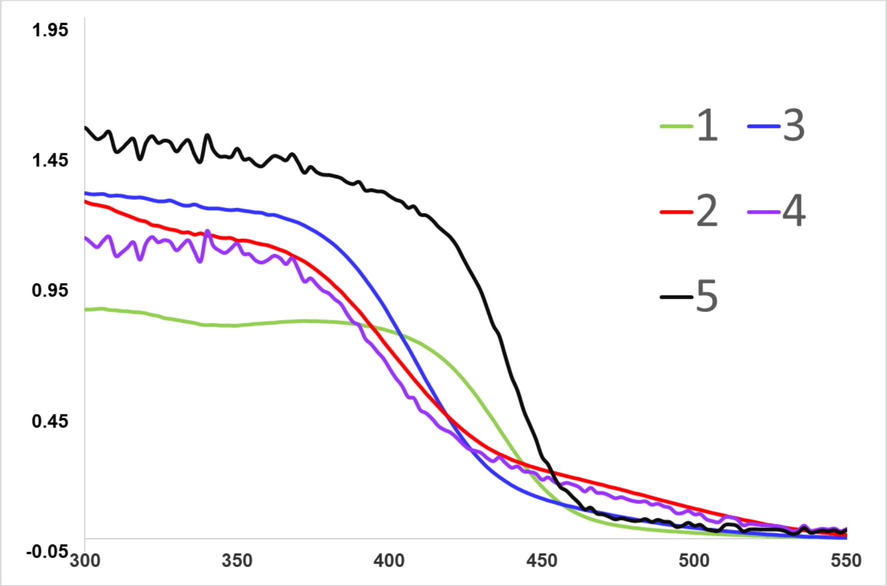

Figure S6. UV-vis spectra of complexes **1-5** in the solid state.

Table S4. The hole-electron analysis of 4 lowest spin forbidden $S_0 \rightarrow T_x$ transitions. The impacts of fragments to the ground and excited states are shown in percents.

1

			Ground state (HOLE)					Excited state (ELECTRON)				
	E, eV	λ, nm	Cu	P2	ligand	PhPz	BF4	Cu	P2	ligand	PhPz	BF4
T1	3.144	394	6	2	0	92	0	1	0	0	98	0
T2	3.464	358	10	14	75	1	0	2	11	87	0	0
T3	3.476	357	5	2	1	92	0	0	0	0	99	0
T4	3.532	351	36	18	14	32	0	2	1	8	88	0

2

			Ground state (HOLE)					Excited state (ELECTRON)				
state	E, eV	λ, nm	Cu	P2	ligand	PhPz	BF4	Cu	P2	ligand	PhPz	BF4
T1	3.146	394	4	1	0	95	0	1	0	0	99	0
T2	3.466	358	2	1	-1	99	0	0	0	0	99	0
T3	3.538	350	25	12	46	17	0	2	3	43	53	0
T4	3.542	350	17	10	61	12	0	1	3	60	35	0

3

			Ground state (HOLE)					Excited state (ELECTRON)				
	E, eV	λ, nm	Cu	P2	ligand	PhPz	BF4	Cu	P2	ligand	PhPz	BF4
T1	3.188	389	32	11	5	52	0	2	1	0	97	0

T2	3.349	370	22	7	3	69	0	1	1	0	98	0
T3	3.530	351	4	1	9	86	0	0	1	11	88	0
T4	3.535	351	2	4	79	16	0	1	4	79	16	0

5

			Ground state (HOLE)					Excited state (ELECTRON)				
	E, eV	λ, nm	Cu	P2	ligand	PhPz	BF4	Cu	P2	ligand	PhPz	BF4
T1	2.585	480	1	1	98	0	0	1	2	97	0	0
T2	2.629	472	0	2	98	0	0	1	2	98	0	0
T3	3.177	390	9	3	1	87	0	1	1	1	98	0
T4	3.412	363	45	17	6	31	0	2	1	1	95	0

Cu – copper atom, P2 – two P atoms, ligand – organic pert of P^P ligand, PhPz – N^N ligand, BF_4 – counterion.

Color codes – green >90%, blue 30-90%

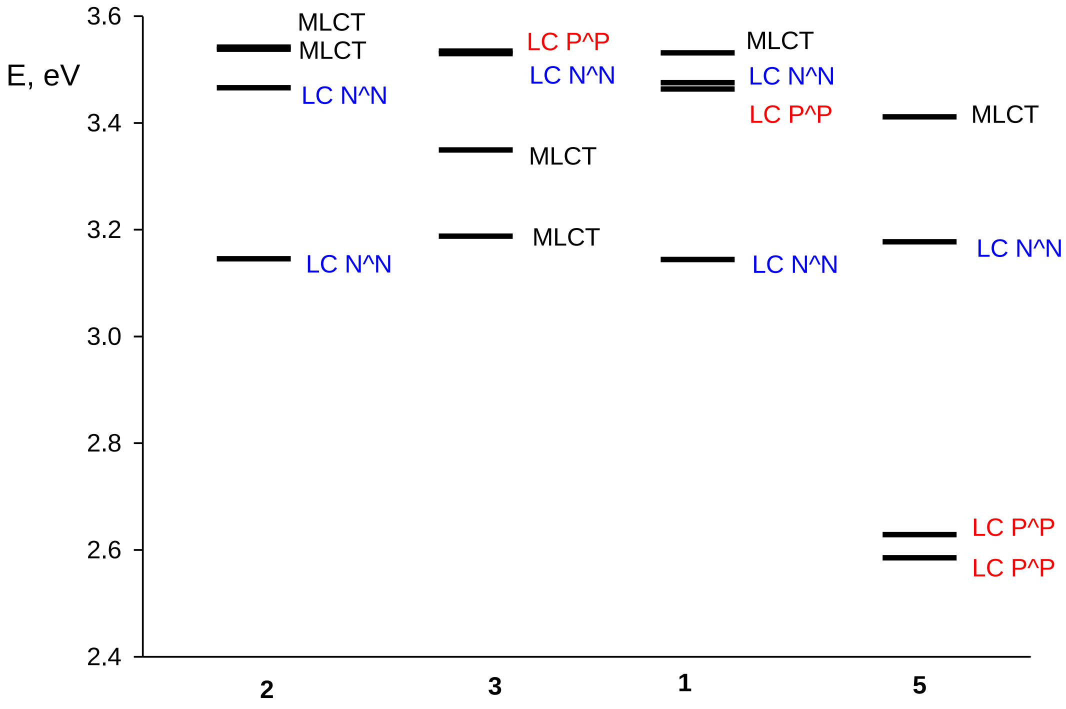

Figure S7. Graphical representation of energies of TDDFT computed spin forbidden $S_0 \rightarrow T_x$ transitions for 1-3, 5. Black – MLCT states, red – P^P ligand centered states, blue N^N ligand centered states. Since excitation pathway is of MLCT nature the MLCT triplet state would be preferred until the alternative state becomes extremely profitable.

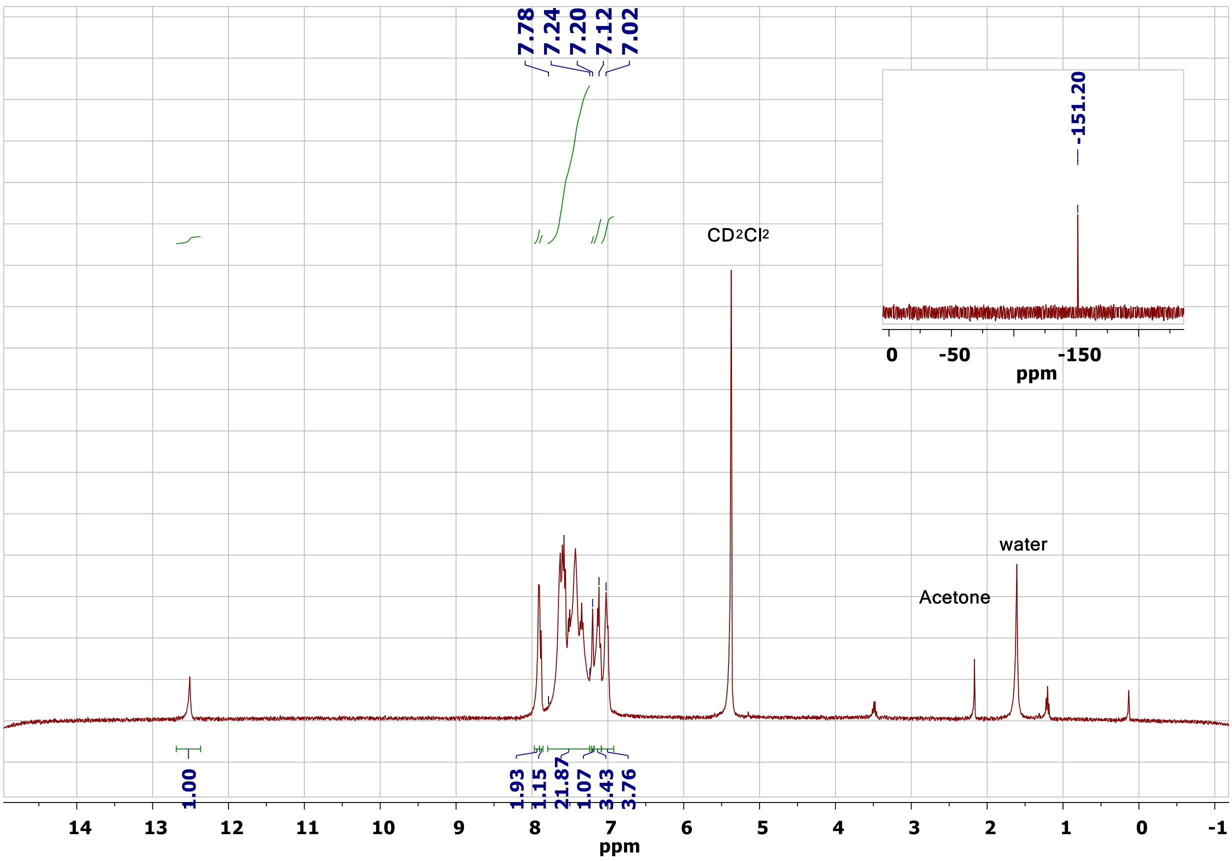

Figure S8. ^{1}H NMR spectrum of complex **1** in CD_2Cl_2, inset ^{19}F spectrum.

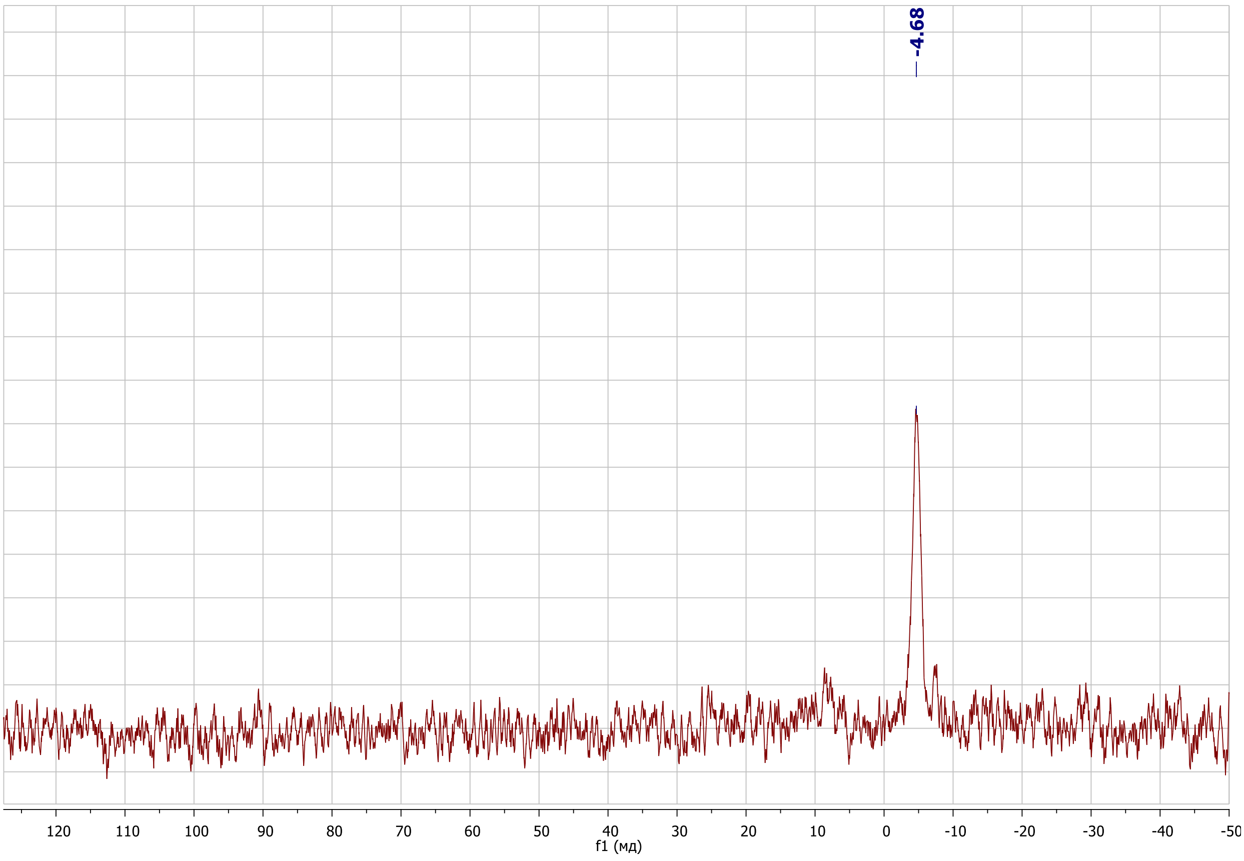

Figure S9. ^{31}P{^{1}H} NMR spectrum of complex **1** in CD_2Cl_2.

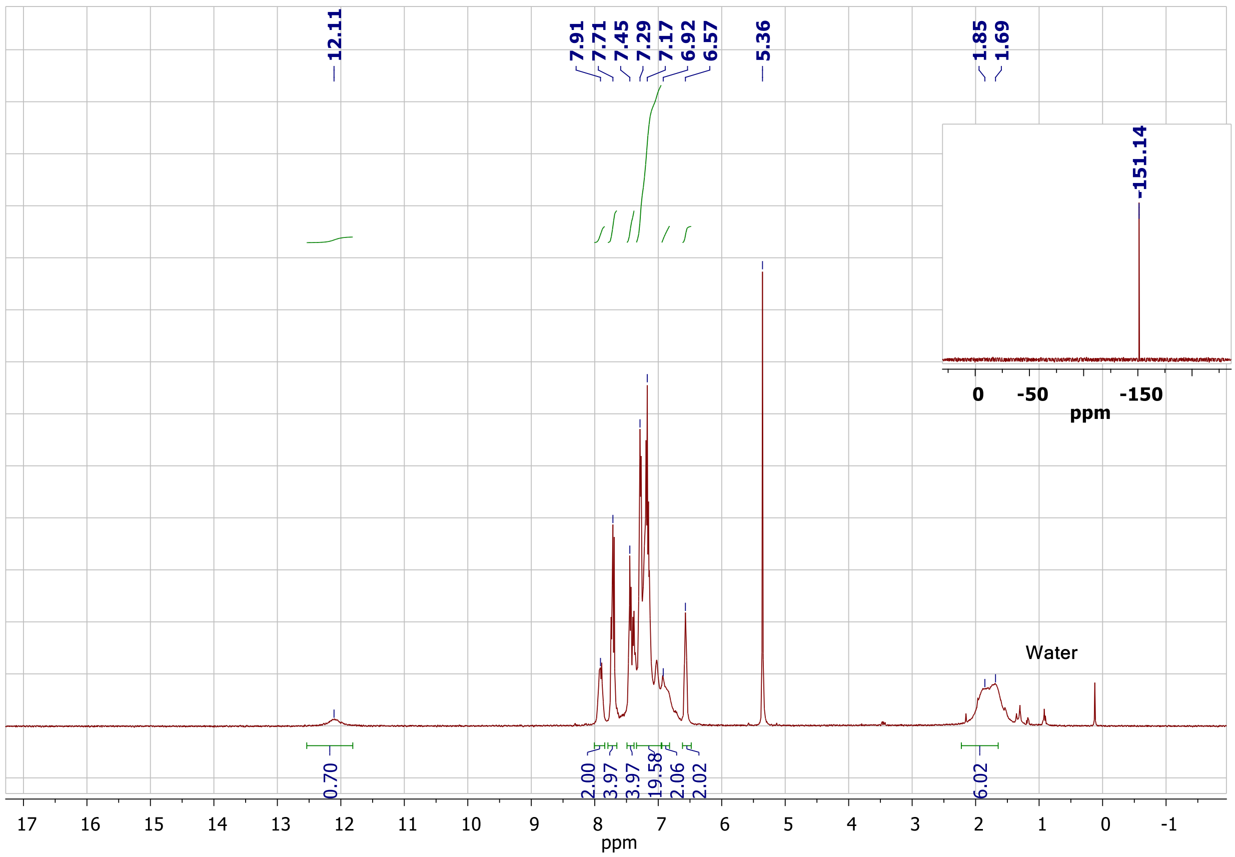

Figure S10. ^{1}H NMR spectrum of complex **2** in CD_2Cl_2, inset ^{19}F spectrum.

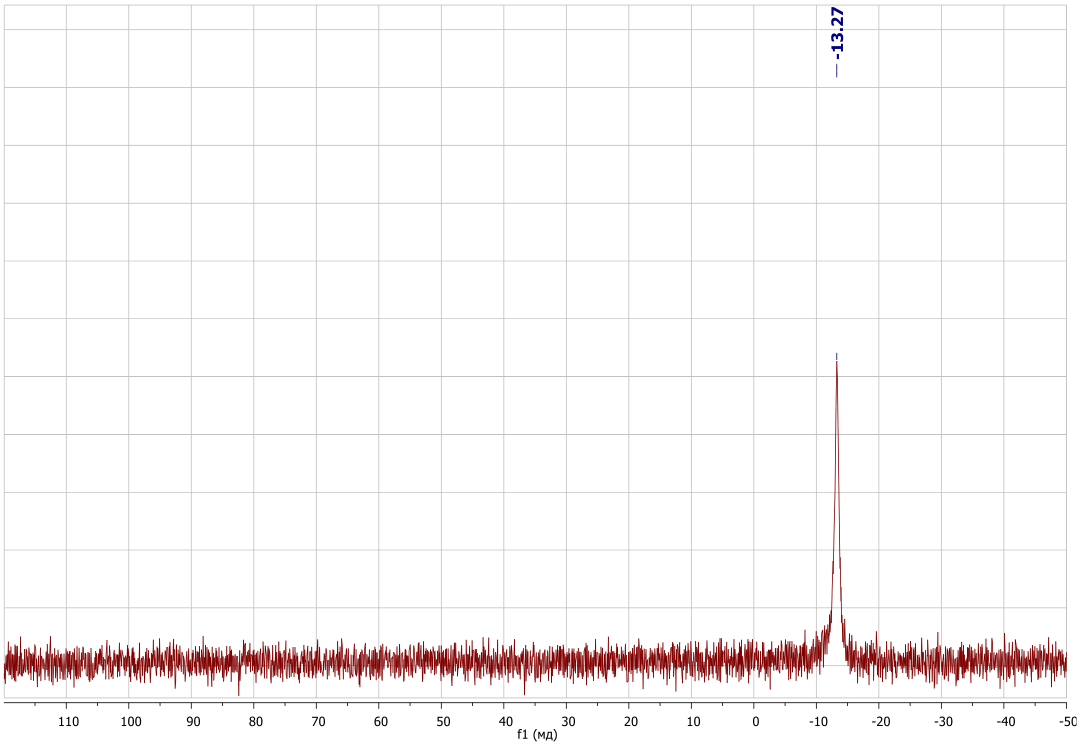

Figure S11. $^{31}P\{^{1}H\}$ NMR spectrum of complex **2** in CD_2Cl_2.

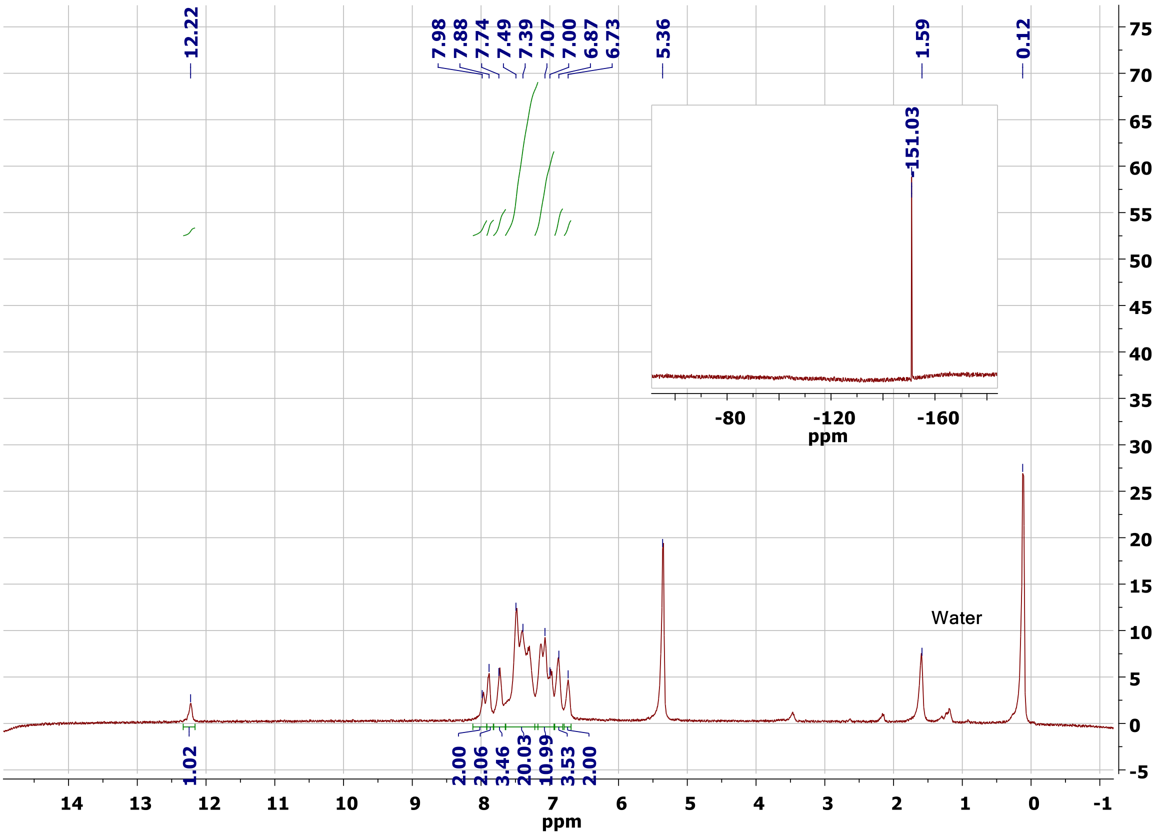

Figure S12. ^{1}H NMR spectrum of complex **3** in CD_2Cl_2, inset ^{19}F spectrum.

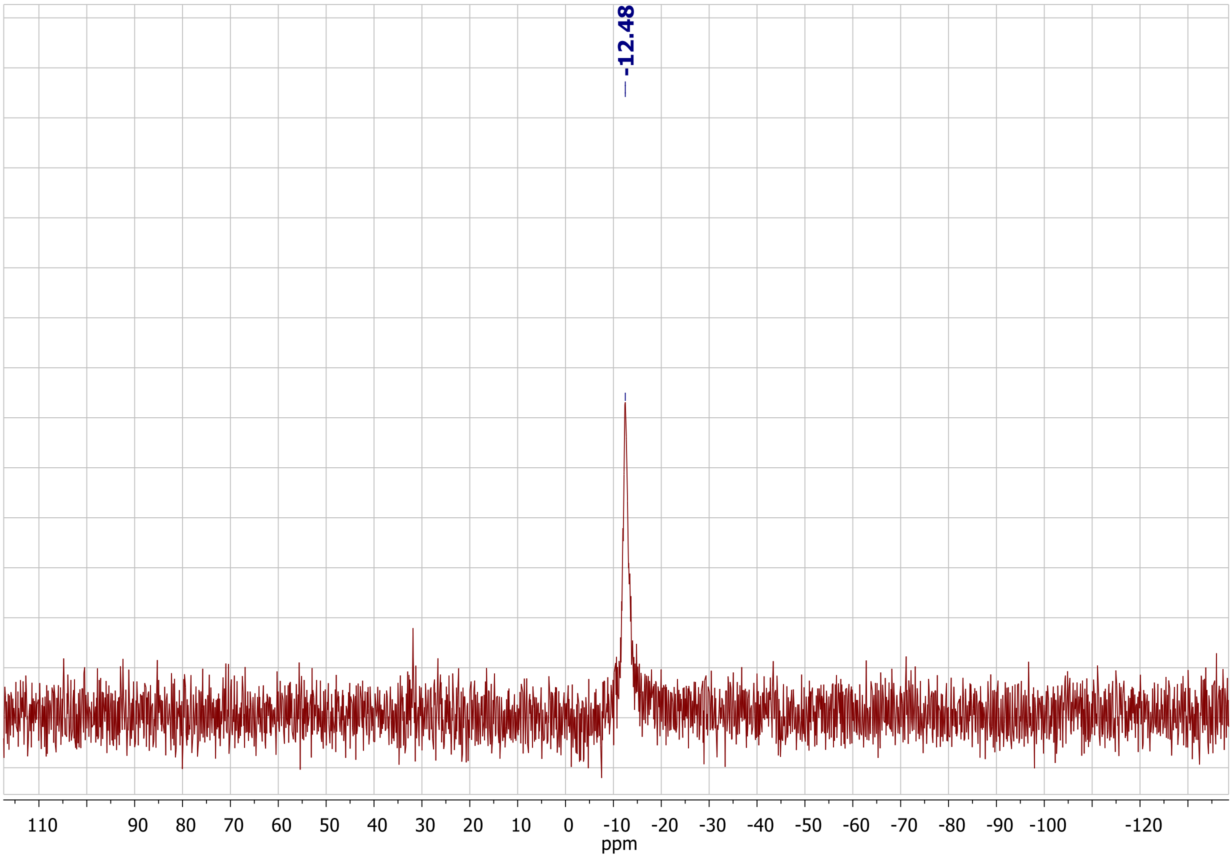

Figure S13. $^{31}P\{^{1}H\}$ NMR spectrum of complex **3** in CD_2Cl_2.

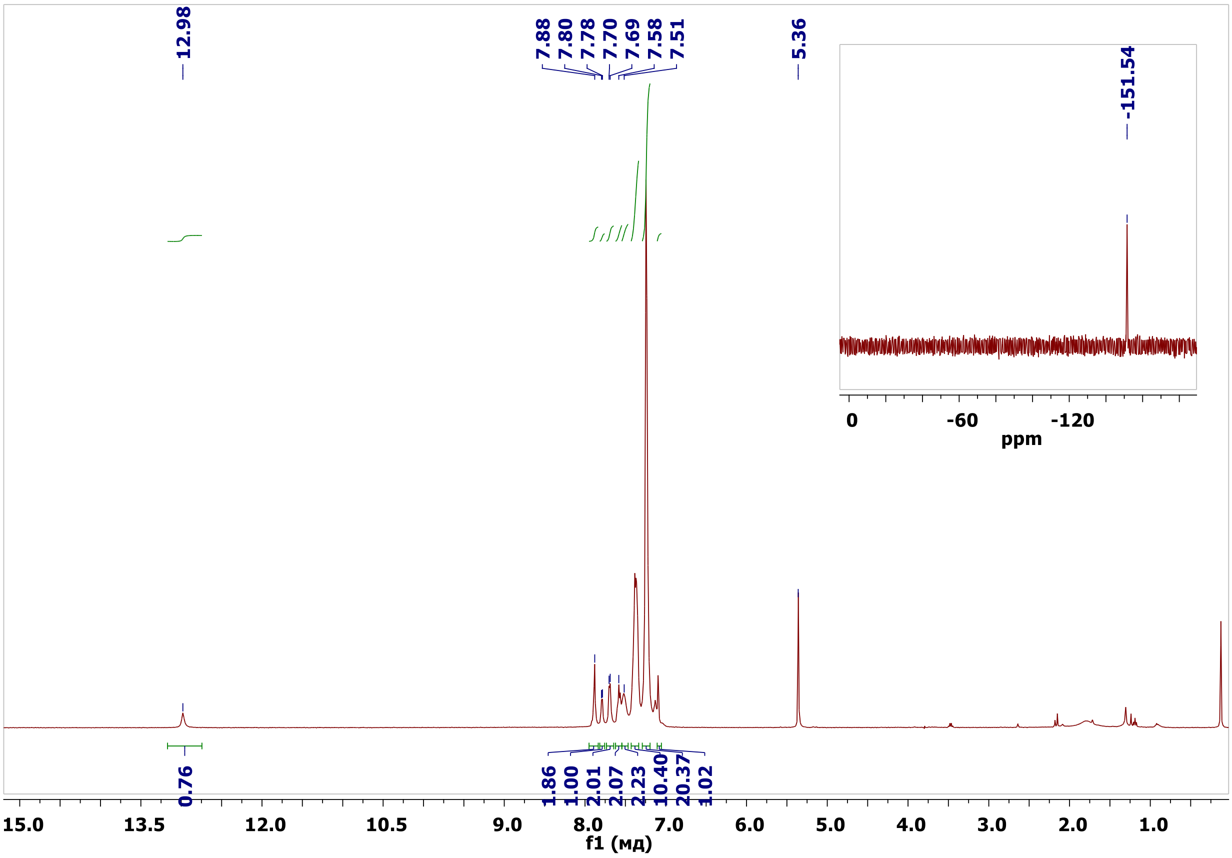

Figure S14. 1H NMR spectrum of complex **4** in CD_2Cl_2, inset 19F spectrum.

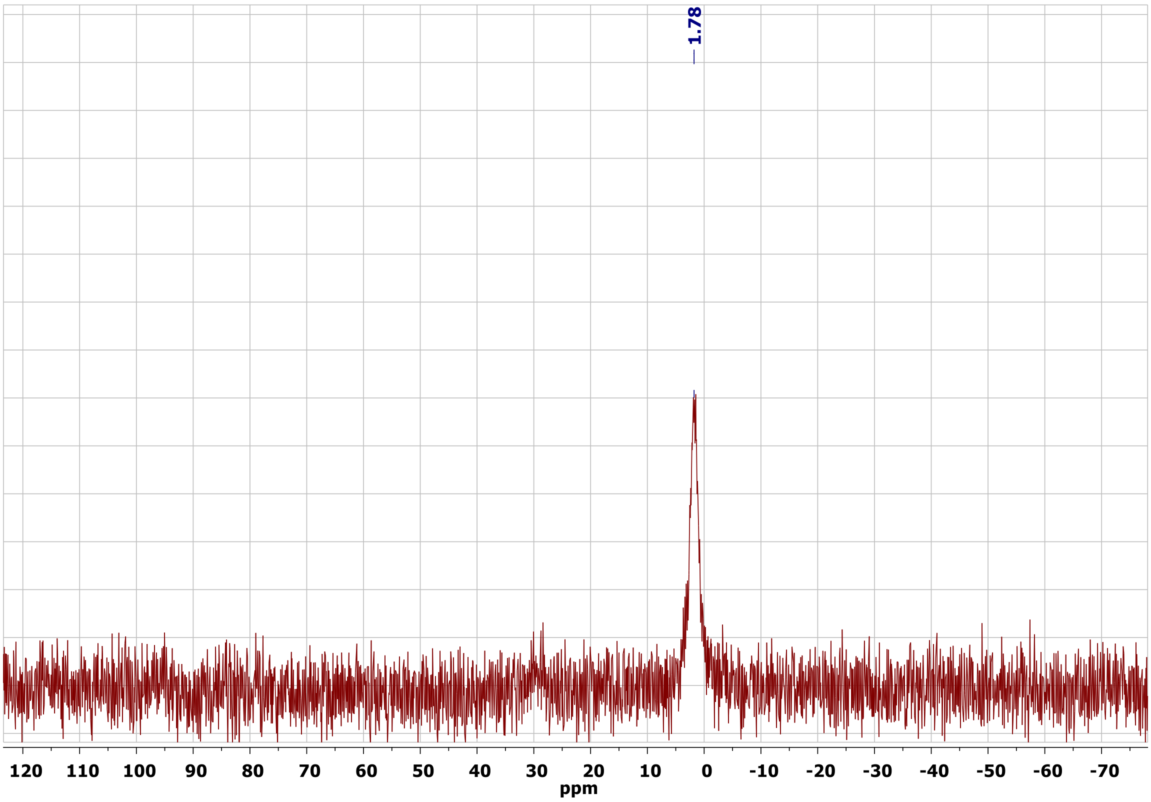

Figure S15. $^{31}P\{^{1}H\}$ NMR spectrum of complex **4** in CD_2Cl_2.

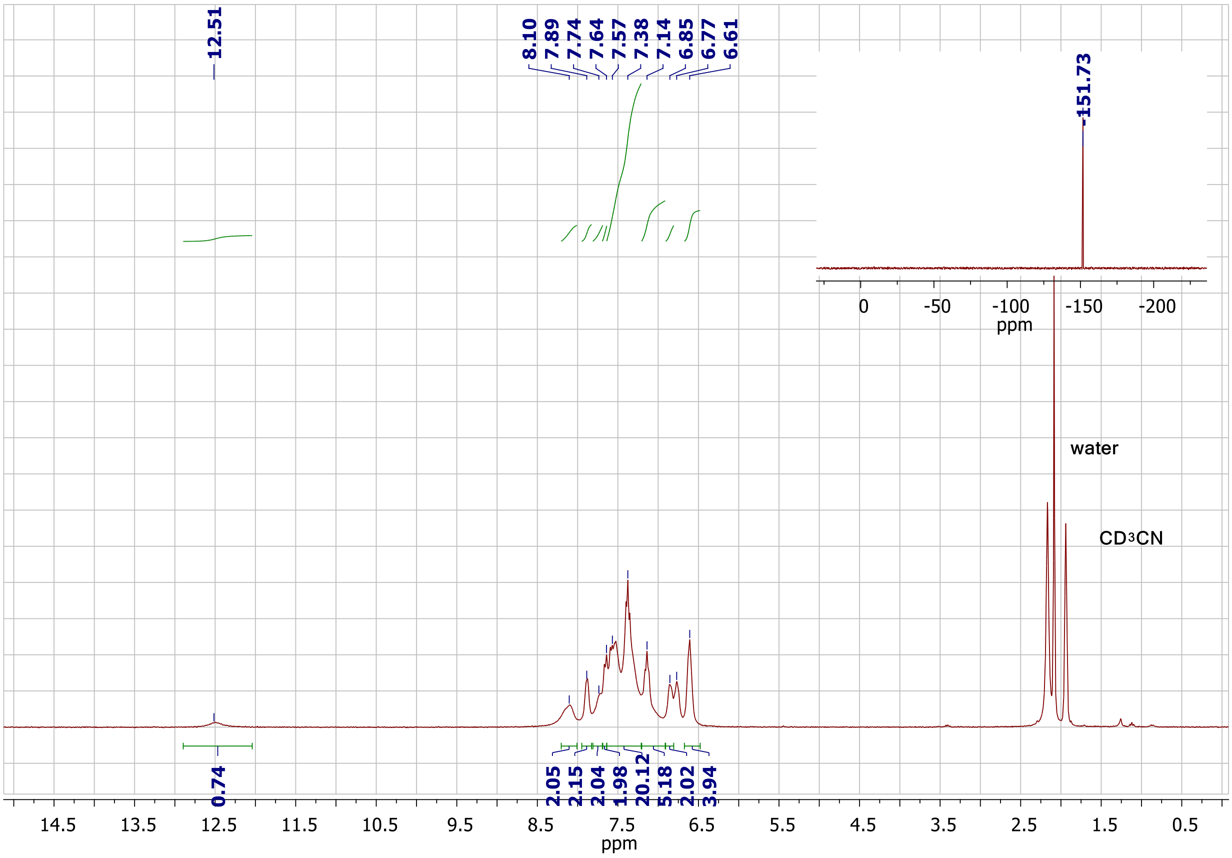

Figure S16. ^{1}H NMR spectrum of complex **5** in CD_3CN, inset ^{19}F spectrum.

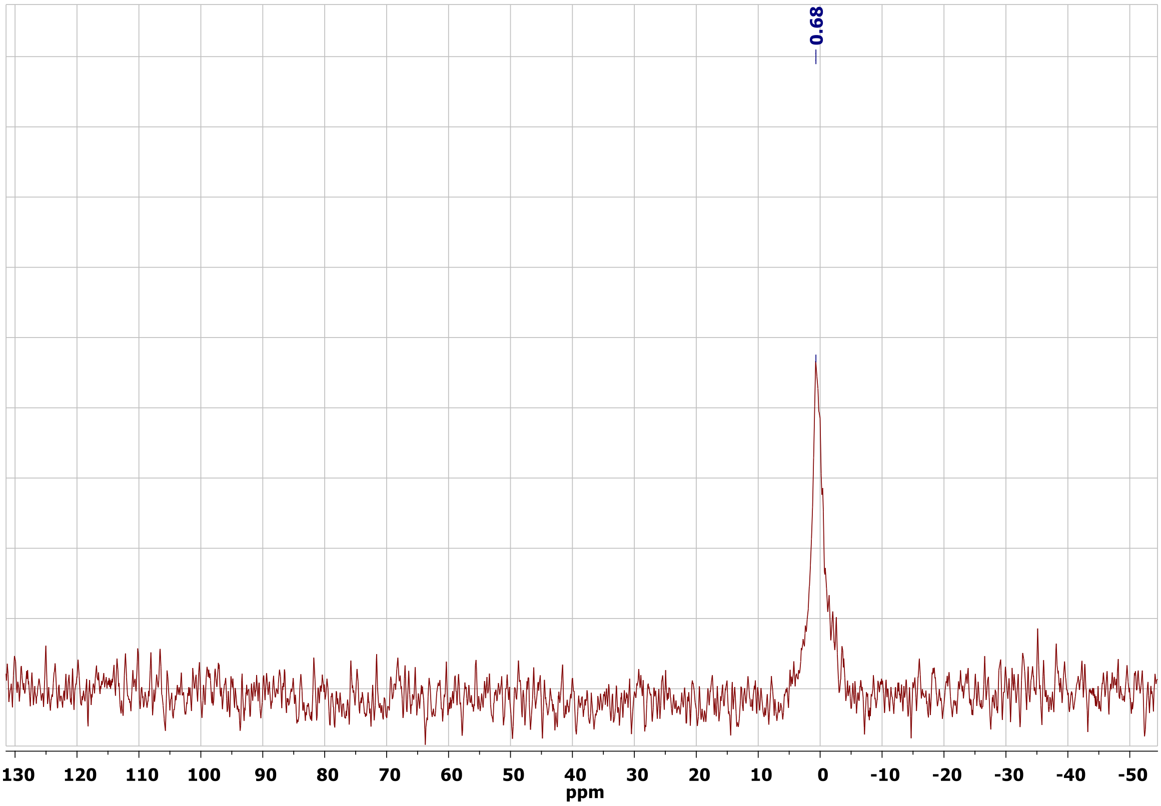

Figure S17. $^{31}P\{^{1}H\}$ NMR spectrum of complex **4** in CD_2Cl_2.

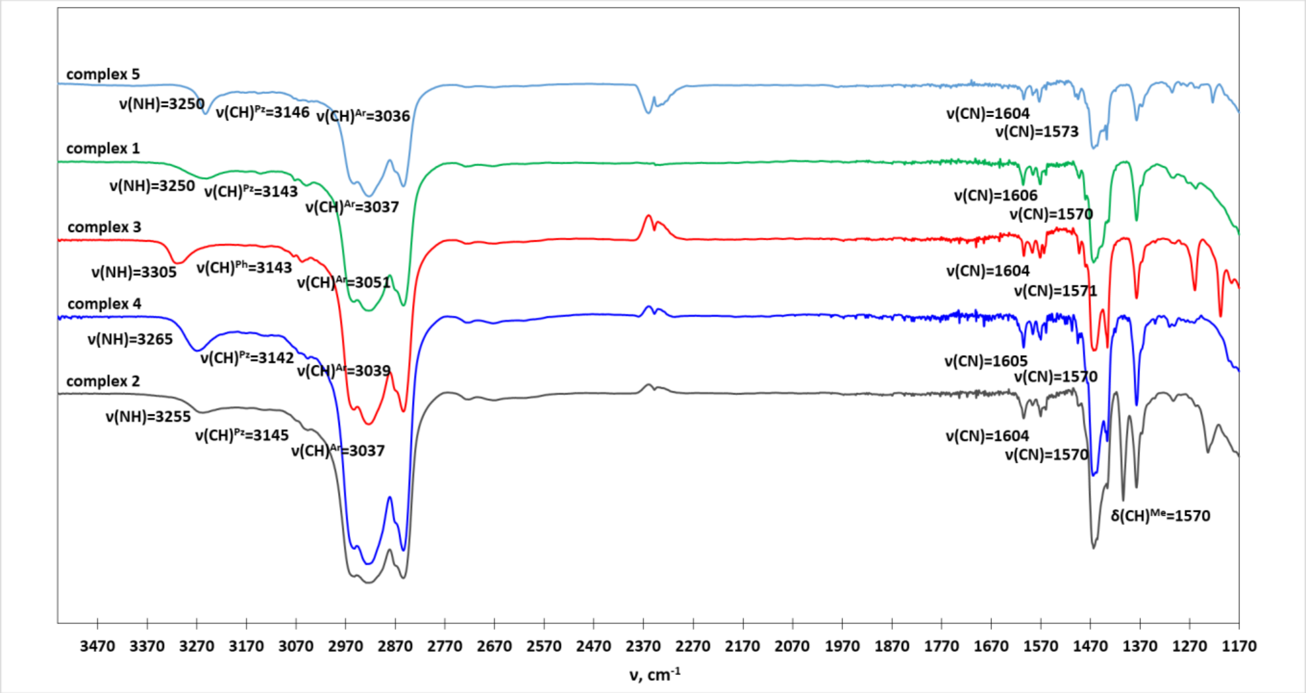

Figure S18. IR spectra of complexes in nujol.
